# Fish introductions in the former Soviet Union: The Sevan trout (*Salmo ischchan*) — 80 years later

**DOI:** 10.1371/journal.pone.0180605

**Published:** 2017-07-06

**Authors:** Wiesław Bogdanowicz, Robert Rutkowski, Bardukh K. Gabrielyan, Akylbek Ryspaev, Anzhela N. Asatryan, Jon A. Mkrtchyan, Barbara M. Bujalska

**Affiliations:** 1 Museum and Institute of Zoology, Polish Academy of Sciences, Warszawa, Poland; 2 Scientific Center of Zoology and Hydroecology, National Academy of Sciences of Armenia, Yerevan, Armenia; 3 Institute of Biology and Pedology, Kirghizstan National Academy of Sciences, Bishkek, Kirghizstan; 4 Sevani Ishkhan CJSC, Yerevan, Armenia; University of Arkansas Fayetteville, UNITED STATES

## Abstract

The Soviet Union played the leading role in fish introductions in Eurasia. However, only 3% of all introductions prior to 1978 gave a commercial benefit. One of the noteworthy examples appears to be the Sevan trout (*Salmo ischchan* Kessler, 1877)—an endemic salmonid of Lake Sevan in Armenia. This species has been introduced to Kirghizstan, Kazakhstan, and Uzbekistan, however, only the Kirghiz population has persisted in relatively high numbers. In this paper we provide the first extensive molecular study of *S*. *ischchan* using samples from the native population from Lake Sevan and three hatcheries in Armenia, as well as from the population introduced to Lake Issyk Kul in Kirghizstan. The Kirghiz population has been isolated since the introductions took place in 1930 and 1936. Our results, based on 11 nuclear microsatellites and a 905 bp fragment of the mitochondrial control region suggest that hatcheries have maintained genetic variability by way of ongoing translocations of individuals from Lake Sevan. Simultaneously, significant Garza-Williamson *M*-values suggest that bottlenecks could have reduced the genetic variability of the wild populations in the past. This hypothesis is supported by historical data, indicating highly manipulated water-level regulations and poaching as two main factors that dramatically impact fish abundance in the lake. On the other hand, a similar situation has been observed in Kirghizstan, but this population likely rebounded from small population size faster than the other populations examined. The Kirghiz population is significantly genetically differentiated from the other groups and have morphological features and biological attributes not observed in the source population. Genetic data imply that the effective population size in the native population is lower than that found in the introduced population, suggesting that some active protection of the Lake Sevan population may be needed urgently.

## Introduction

The Soviet Union, with its almost 1,400 bodies of water (of which 87% were natural lakes, 7% artificial reservoirs and 6% rivers) played the leading role in fish introductions in Eurasia [1]. According to Burmakin [2] approximately 51 species of freshwater fish from 12 families were introduced into Soviet bodies of water up until1957. Within its European territory alone, there were 70 species (40 exotic and 30 native) introduced until, and transferred among these bodies of water, until the end of 1980s, i.e. slightly more than half of the total number of fish species received by all other European countries at that time. The motives for these introductions varied from species to species, but in general, improvement of the wild stock (e.g., establishing or restoring fisheries, stocking natural waters, providing food for predators, establishing a wild stock and controlling stunted species [3]) was the primary reason for introductions, followed by aquaculture [1]. However, only 3% of all introductions released in the Soviet Union prior to 1978 gave a commercial benefit [4], and one of the successful examples appears to be the Sevan trout—*Salmo ischchan* Kessler, 1877.

**Fig 1:**
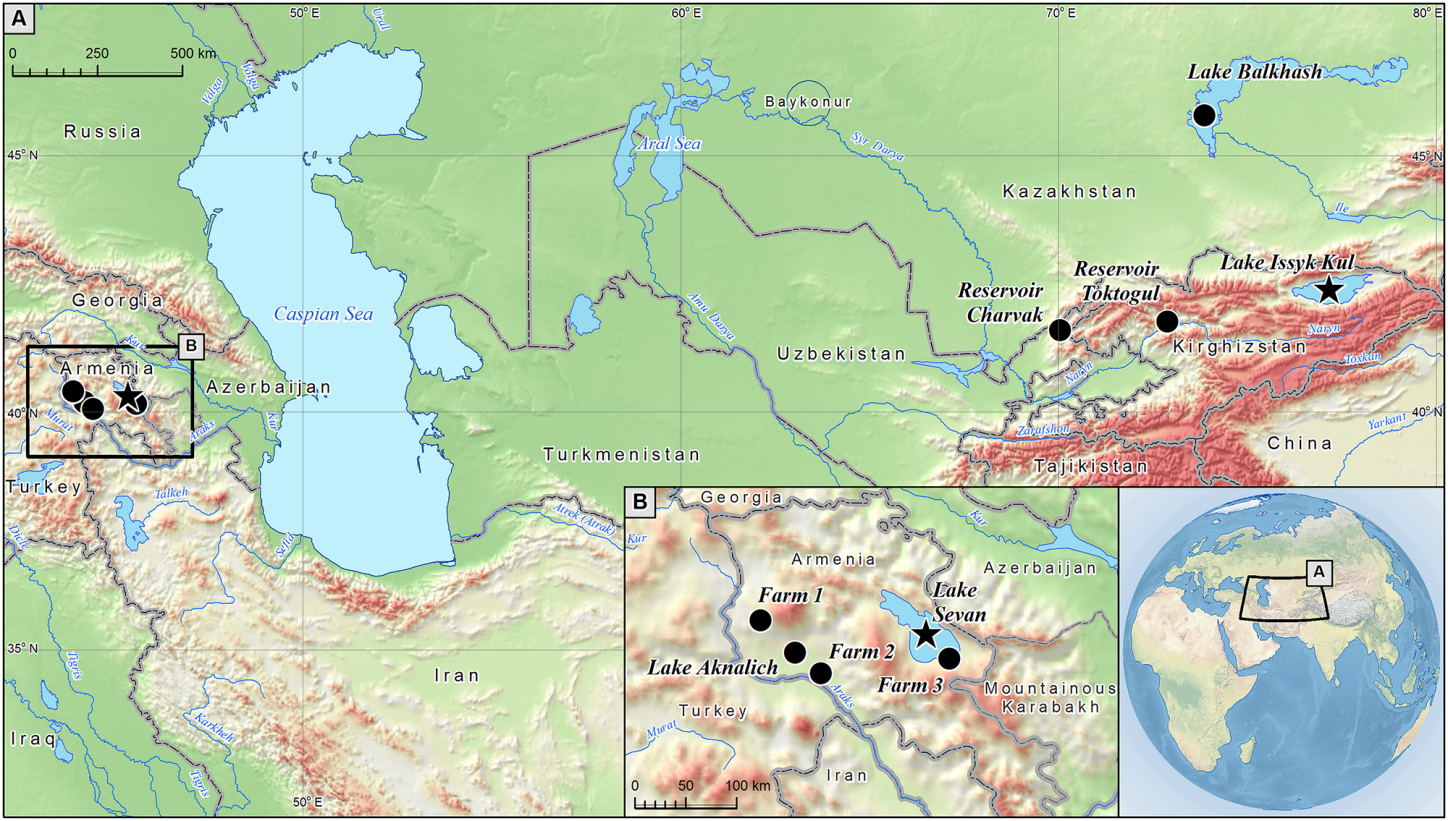
The map of the Caucasus region and Central Asia, where two wild populations of Sevan trout were sampled. Stars refer to populations under study living in the wild, black circles show other water bodies (including three analyzed farms), where the species was introduced.

The Sevan trout is an endemic salmonid species (e.g., http://www.fishbase.org/; cf. [5, 6]) occurring in Lake Sevan in Armenia (Fig 1), closely related to the brown trout (*Salmo trutta*) [7]. Some authors, following a conservative approach to taxonomy, even treat it as a variant of the highly variable Danube lineage of *S*. *trutta* (e.g., [8]). Within the Armenian territory this species is also present in Lake Aknalich, where it was first introduced from Lake Sevan in 1966–1974. Its population in Lake Aknalich is maintained by annual restocking for the sole purpose of production, and *S*. *ischchan* would not otherwise survive there. In the last few years broodstock in this lake has been stocked with fish originating from different hatcheries, mainly from the Ararat valley (B.K. Gabrielyan, pers. comm.). Two other fish species endemic to Lake Sevan are the Sevan khramicarp (*Varicorhinus capoeta sevangi*) and Sevan barbel (*Barbus goktchaicus*).

The Sevan trout was divided into four distinct subspecies (morphs) differing in terms of breeding times and places, as well as growth rate: the winter bakhtak (*S*. *i*. *ischchan*); the summer bakhtak (*S*. *i*. *aestivalis*); the gegarkuni (*S*. *i*. *gegarkuni*); and the bodjak (*S*. *i*. *danilewskii*). Two of these subspecies bred exclusively in the lake: the winter bakhtak (the largest form, growing up to 90 cm in total length and reaching 15 kg) and the bodjak (dwarf, slowly-growing lacustrine fish, not exceeding 33 cm in total length and 0.25 kg). Unfortunately, although the winter bakhtak and the bodjak are listed in the Red Data Book of Armenia, they are most likely now extinct [9]. At present, only two subspecies occur in the native area: the summer bakhtak and the gegarkuni, both usually less than 60 cm in body length and 4 kg in body mass. The former breeds naturally in rivers and within the lake near river mouths. The latter is in turn a migratory form that breeds exclusively in rivers.

In the former Soviet Union the Sevan trout was a promising subject for acclimatization in other bodies of water. The stocking of Lake Issyk Kul in Kirghizstan with *S*. *i*. *gegarkuni* serves as a good example of this process. Over 1.5 million fertilized eggs were transferred, and over one million fry of 19 to 24 mm in length (i.e. ages of 22 to 45 days) were introduced into that lake in 1930 and 1936 [10] in order to start a commercial trout fishery. After introduction to its new environment, the biology and morphology of the Sevan trout changed so markedly that it began to be treated as a new subspecies, *S*. *i*. *issykogegarkuni* Lushin, 1951 [10, 11]. This species was also introduced to Lake Balkhash in Kazakhstan in 1971 and 1974 (1.3 million larvae) and to Reservoir Charvak in Uzbekistan in 1973–1983 (over 3 million fry). Both samples originated from Issyk Kul, Kirghizstan ([12]; E.E. Khurshut, in litt.; see also [13]) as the sample (3.6 million larvae) used in the late 1970s for stocking Reservoir Toktogul in Kirghizstan [14], where the Sevan trout now naturally breeds (A. Ryspaev, pers. comm.; Fig 1). The Uzbek population in the wild appears to be well established according to the Introduced Species Fact Sheets of FAO Fisheries and Aquaculture Department [15]. In contrast, the introduction to Kazakhstan was unsuccessful and the species is no longer listed among fishes occurring in the country [16].

The objective of our study was to genetically evaluate the documented history of the introduction of *S*. *ischchan* into Lake Issyk Kul in Kirghizstan using a suite of microsatellite markers in combination with a mitochondrial marker. We aimed to evaluate levels of genetic diversity of two introduced populations in relation to the native population. We also attempted to estimate the ‘genetic quality’ of the stock material in three fish farms in Armenia. We hypothesize that due to the more limited gene pool the fish stock on farms should exhibit lower genetic variability than natural populations. Similarly, we expect that in breeding stocks, genetic effects of the founder event should be detected. We also hypothesize that the introduced population from Kirghizstan should have a lower level of genetic variability and, like the breeding stocks, exhibit genetic signatures of past demographic bottlenecks. As the population from Kirghizstan has been isolated for many years, we also expect that genetic drift and selection should differentiate this fish from the source population in terms of the frequency of microsatellite alleles, whereas the population from Lake Sevan and the three investigated breeding stocks should show a lower level of genetic differentiation because individuals are still being transferred among those sites.

## Materials and methods

We sampled gegarkuni individuals from two regions, the native range in Lake Sevan, Armenia (native population called *Wild1*, *N* = 33), and the introduced range in the region of Lake Issyk Kul, Kirghizstan (naturalized population called *Wild2*, *N* = 61), more than 2,000 km away from each other (Fig 1), to compare the genetic characteristics and relationships between populations. In addition, we sampled gegarkuni from three fish farms situated in Armenia: Akunk in the Aragatsotn Province (*N* = 23), Masis in the Ararat plain (*N* = 29) and Karchakhpyur in the Gegharkunik Province (*N* = 116), denoted as *Farm1*, *Farm2* and *Farm3*, respectively, to compare with the wild populations. All farms are being used to ensure the survival of large numbers of fry. In total, we analyzed 262 samples—all were collected in 2010, 2011 and 2015.

Lake Sevan is the largest lake in Armenia and the Caucasus region. It is one of the largest fresh-water high-altitude lakes in the world, located at an elevation of 1,916 m above sea level [17]. The total surface area of its basin is about 5,000 km^2^; the lake itself is 1,240 km^2^ (B.K. Gabrielyan, in litt.). Before human intervention dramatically changed the Lake Sevan ecosystem, the lake had the maximum depth of 95 m, covered an area of 1,360 km^2^ and had a perimeter of 260 km [18]. Lake Issyk Kul is situated at the altitude of 1,621 m above sea level in the northeastern part of the Kirghizstan. This lake is the second largest high-altitude lake in the world, having 6,206 km^2^ of water surface area, 184 km in length, and 60 km in width. Its depth averages 279 m but the maximum is 702 m [17].

### Laboratory protocols

#### Microsatellite markers

DNA was isolated from tissue samples (usually small pieces of fins) collected in Armenia and Kirghizstan. Genomic DNA was extracted using Nucleic Acid Isolation System QuickGene-810 (Fujifilm) with standard protocol. Eleven pairs of primers were used to amplify microsatellite loci. All of these primers were described previously by Nikolic et al. [19] for the Atlantic salmon (*Salmo salar*), but annealing temperatures were modified (Table 1). Primers were fluorescently labelled by Oligomery WellRED dyes (D2, D3 and D4) recommended by Beckman Coulter. In the case of loci BHMS328, BHMS230, BHMS176, BHMS304-1, SSsp2216, SsaD157 and BHMS176A, PCR was performed in a total volume of 10 μl containing 1 μl of template DNA, 0.2 μl (0.2 μM) of each primer and 5 μl of Multiplex PCR Master Mix (Qiagen). The thermal profile for SSsp 2216, SsaD 157 and BHMS176A was 95°C/15 min, 35 cycles—94°C/30 s, 57°C/1.5 min, 72°C/1.5 min and final extension in 60°C/30 min. For the remaining loci the annealing temperature was changed to 56°C. Non-multiplex genotyping was performed for Ssa197, BHMS259, BHMS217 and BHMS429 using 7.5 μl of polymerase REDTaq^®^ReadyMIX^™^ PCR Reaction Mix with MgCl_2_ (Sigma-Aldrich), 0.5 μl of labeled forward primer (5 μM), 0.5 μl of reverse primer (5 μM) and 2μl of genomic DNA. Amplification conditions were 94°C/3 min, 35 cycles—94°C/40 s, primer-specific elongation (see Table 1)/50 s, 72°C/50 s and final extension of 72°C/15 min. Amplified microsatellite products were genotyped in an CEQ^™^8000 sequencer (Beckman Coulter) and analyzed using Beckman Coulter Fragment Analysis Software. Two-digit genotypes of individuals of *S*. *ischchan* at 11 microsatellite loci are shown in S1 Table.

**Table 1:** Results of cross-amplification of 11 microsatellite loci in *S*. *ischchan* (*n* = 262). TA—optimal annealing temperature for PCR; size range—size of fragments obtained during PCR; *A*—number of alleles; *H*_O_—heterozygosity observed; *H*_E_—heterozygosity expected; *HWE*—*P-*values for HWE exact test for heterozygote deficiency/excess; *F*_IS_—fixation index; ^F^—significant after FDR correction; *PIC*—polymorphic information content for a given locus.

Locus	TA	Size range	*A*	*H*_O_	*H*_E_	*HWE*	*F*_IS_	*PIC*
Ssa197	54	111–147	10	0.782	0.830	0.0003^F^	0.059	0.809
BHMS259	52	108–124	7	0.359	0.369	0.1492	0.029	0.349
BHMS217	52	231–239	4	0.435	0.481	0.3249	0.097	0.417
BHMS429	54	191–201	5	0.146	0.164	0.1548	0.112	0.156
BHMS328	56	120–130	6	0.398	0.428	0.0062^F^	0.072	0.387
BHMS230	56	367–419	20	0.710	0.763	0.0001^F^	0.072	0.738
BHMS176	56	119–131	6	0.244	0.290	0.0125^F^	0.159	0.274
BHMS304-1	54	137–191	15	0.531	0.605	0.0001^F^	0.125^F^	0.572
SSsp2216	60	139–227	20	0.797	0.818	0.0001^F^	0.028	0.794
SsaD157	57	234–392	34	0.847	0.917	0.0001^F^	0.078	0.911
BHMS176A	57	160–162	2	0.187	0.245	0.0003^F^	0.239^F^	0.215
Mean/overall			11.73	0.494	0.537	0.0001	0.082	

#### Mitochondrial DNA

Amplification of control region fragment was performed with primers PST (forward) 5’CCCAAAGCTAAAATTCTAAAT and FST (reverse) 5’GCTTTAGTTAAGCTACGC described by Cortey and García-Marín [20]. The PCR reaction mixture (total volume—50μl) consisted of polymerase REDTaq^®^ReadyMIX^™^ PCR Reaction Mix with MgCl_2_ (Sigma-Aldrich), both primers of concentration 0.2 μM and 3μl of template DNA. The amplification was performed in a Veriti^®^ Thermal Cycler (Applied Biosystems) under the following conditions: 94°C/3 min—initial denaturation, 40 cycles—94°C/1 min, 52°C/1 min, 72°C/1 min, and final extension of 72°C/5 minutes. The amplification products were separated on 1.5% agarose gel, and then purified using the purification kit Clean-Up (A&A Biotechnology). Sequencing reactions were performed using a BigDye Terminator v3.1 Cycle Sequencing Kit (Life Technology). Detection of sequencing reaction products was carried out on 3500xL Genetic Analyzer (Applied Biosystem). Although the designed primers amplified a fragment of about 1,000 base pairs (bp), only 905 bp were clearly readable, due to a presence of mononucleotide (poliT) fragment between positions corresponding to nucleotide 561 and 563 of the Atlantic brown trout complete control region sequence (GenBank accession no. AF253541.1). All sequences generated were submitted to GenBank (accession nos. KY448292–KY448302).

### Statistical analysis

#### Microsatellites

Possible problems with null alleles, genotyping errors, and large allele drop-out were diagnosed using MICROCHECKER v. 2.2.3 [21]. To estimate frequency of null alleles we used ML-NullFreq [22]. We performed 100,000 randomizations.

Microsatellite polymorphism was estimated at three levels. First, we assessed allelic diversity (*A*), allelic richness (*R*—[23]), mean number of private alleles (*P*), private allelic richness (R_P_), effective number of alleles (*E*_*A*_), observed heterozygosity (*H*_O_) and unbiased expected heterozygosity (*H*_E_) [24], for all samples to describe the results of cross-species amplification. A fixation index (*F*_IS_) for each locus was calculated and its significance was tested under randomization procedure. Multiple comparisons were corrected using the False Discovery Rate (FDR) correction [25], as advocated by Narum [26]. These analyses were performed using GenAlEx version 6 [27], FSTAT version 2.9.3 [28] and HP-RARE [29]. Using MSTool [30] we estimated the polymorphic information content (PIC index) for each locus. Genotypic linkage disequilibrium among all pairs of loci, as well as a probability test for deviation from Hardy-Weinberg equilibrium was evaluated using Genepop on the Web version 4.0.10 [31, 32].

Genetic differentiation between populations was estimated using *F*_ST_. Overall *F*_ST_ [33] for wild and farm populations and pairwise *F*_ST_ among all the populations studied were obtained using FSTAT. The 95% confidence intervals for overall *F*_ST_ were also estimated in FSTAT. We also used the BAYESASS 3.03 program [34], with default and adjusted settings, to assess recent migration rates and the direction assured by migration among sites. We adjusted mixing parameters for allele frequencies (Δ_*A*_), inbreeding coefficients (Δ_*F*_) and migration rates (Δ_*M*_) so that the acceptance rates were 38%, 45%, and 44%, respectively. MCMC was run for 10,000,000 steps, with 1,000,000 steps discarded as burn-in, and with sampling every 100^th^ step. Convergence and mixing were inspected visually using Tracer v 1.5 [35], with effective sample sizes greater than 100 assured for all the recorded parameters.

To deduce the demographic history of the populations we attempted to identify possible genetic effects of fluctuations in the effective population size, following introductions and farm breeding practices. First, we tested for heterozygosity excess using the BOTTLENECK, ver. 1.2.02 program [36]. The two-phase mutation (TPM) model with 10% multistep mutations was applied [37]. We used a Wilcoxon signed rank test to determine which population had significant heterozygote excess across loci. Subsequently we evaluated whether or not the allele frequency was normal (L-shaped), suggesting stability of effective population size or shifted, i.e., indicating bottleneck. We also tested for a bottleneck signature using *M*-ratio [38]. In this method the ratio of the total number of alleles to the range of allele sizes is calculated (*M*-ratio) and compared to a critical value determined by simulations as described in [38], with the expectation that the number of alleles declines faster than the allele range in a bottlenecked population. A bottleneck is detected when the observed average *M*-ratio is lower than the critical value (*M*_C_) (defined such that 5% of the simulations fall below *M*_C_). We used M_P_VAL software to calculate *M*-ratio and contrast the observed value to a distribution of M values to calculated values from simulated populations, assumed to be in mutation-drift equilibrium. We used the program Critical_M [38] to calculate *M*_C_ given the observed sample size. These simulations were based on three parameters: *θ*, *p*_g_, Δg. The parameter *θ* = 4 *N*_e_μ, where *N*_e_ = pre-bottleneck effective population size and μ = mutation rate. We used three different population sizes: 100, 500 and 1,000, and a common estimate of microsatellite mutation rate = 0.0005 mutations/generation/locus [39]. The percentage of mutations larger than a single step (*p*_g_) was set to 0.10 and the mean size of mutations larger than a single step (Δ_g_) to 3.5 according to recommendations of Garza and Williamson [38]. Each set of simulations comprised 10,000 iterations. These three tests (heterozygosity excess, allele frequency distribution and *M*-ratio) provide evidence of population decline and recovery over different time scales; the first two indicators are expected to recover relatively quickly, whereas the *M-*ratio has a long recovery time [38].

Bayesian-clustering method (STRUCTURE ver. 2; [40]) was used to examine how well the predefined ‘populations’ corresponded to genetic groups (*K*). STRUCTURE was run 15 times for each user-defined *K* (1–10), with an initial burn-in of 100,000, and 500,000 iterations of the total data set. The admixture model of ancestry and the correlated model of allele frequencies were used. Sampling location was not used as prior information. We then examined Δ*K* statistics that identify the largest change in the estimates of *K* produced by STRUCTURE, as Δ*K* may provide a more realistic estimation of *K* than methods based on likelihoods [41].

To visualize STRUCTURE results we used STRUCTURE HARVESTER [42] and applied CLUMPP [43] to average the multiple runs given by STRUCTURE and to correct for label switching. The output from CLUMPP was visualized with DISTRUCT version 1.1 [44] to display the results.

Relatedness among individuals was estimated using Queller & Goodnight’s [45] *r*-parameter, as implemented in GENALEX 6.41 [27]. Confidence intervals for the population mean at the 95% confidence level were calculated by applying the permutations procedure (1,000 permutations), with 1,000 bootstrap re-samplings.

We estimated the present effective population sizes (*N*_e_), in the farms and wild populations. We used point estimates of *N*_e_, applying the linkage disequilibrium (LD) method, as implemented in NeEstimator version 2.01 [46]. We set the lowest allele frequency to 0.05, as this value allowed for the exclusion of all microsatellite alleles occurring in populations in a single copy (in one heterozygote genotype). However, we also performed calculations with no allele frequency restriction. Additionally, we use the approximate-Bayesian method, implemented in ONESAMP [47]. In this analysis, *N*_e_ for *Farm1* was estimated using 10 loci, as locus BHMS429 was monomorphic in this population (Table 2). For all populations the maximum and minimum effective population sizes were set to 6,000 and 10, respectively.

**Table 2:** Characterization of microsatellite polymorphisms from five populations (*n* = 262). *N—*sample size within population with successful amplification; *A*—number of alleles; *R*_A_—allelic richness; *R*_P_*—*private allelic richness, *H*_O_—heterozygosity observed; *H*_E_—heterozygosity expected; *HWE*—*P-*values for HWE exact test for heterozygote deficiency/excess; NA—not analyzed; in bold—deviation significant at α = 0.05; *F*_*IS*_—fixation index. None of HWE or *F*_IS_ values were significant after FDR correction.

Locus	*N*	*A*	*R*_A_	*R*_P_	*H*_O_	*H*_E_	*HWE*	*F*_IS_
*Farm1*								
Ssa197	23	10	9.81	0.17	0.870	0.811	0.979	-0.050
BHMS259	23	5	4.91	0.91	0.348	0.432	0.085	0.216
BHMS217	23	4	3.99	0.99	0.478	0.583	0.477	0.201
BHMS429	21	1	1.00	0.00	0.000	0.000	NA	NA
BHMS328	23	4	3.99	0.00	0.609	0.615	0.365	0.033
BHMS230	23	9	8.65	3.04	0.652	0.680	0.143	0.063
BHMS176	23	3	3.00	0.00	0.304	0.392	**0.032**	0.245
BHMS304-1	23	9	8.73	2.12	0.652	0.732	**0.016**	0.130
SSsp2216	23	13	12.47	5.21	0.957	0.854	0.396	-0.098
SsaD157	23	23	21.94	5.70	0.870	0.940	**0.031**	0.097
BHMS176A	23	2	2.00	0.00	0.174	0.340	**0.037**	0.506
*Farm2*								
Ssa197	29	8	7.36	0.00	1.000	0.768	0.055	-0.287
BHMS259	29	5	4.70	0.10	0.483	0.406	1.000	-0.172
BHMS217	29	3	3.00	0.00	0.483	0.509	0.625	0.069
BHMS429	29	3	2.72	0.74	0.207	0.188	1.000	-0.080
BHMS328	29	3	3.00	0.00	0.448	0.373	0.782	-0.186
BHMS230	29	8	7.41	1.81	0.621	0.732	**0.010**	0.169
BHMS176	29	4	3.63	0.72	0.207	0.192	1.000	-0.060
BHMS304-1	29	8	7.09	1.04	0.586	0.545	0.898	-0.059
SSsp2216	29	11	10.30	2.63	0.931	0.864	**0.042**	-0.060
SsaD157	29	15	13.38	2.47	0.966	0.882	0.378	-0.077
BHMS176A	29	2	1.72	0.00	0.034	0.034	NA	0.000
*Farm3*								
Ssa197	116	9	7.45	0.01	0.707	0.741	0.069	0.050
BHMS259	116	5	3.35	0.12	0.388	0.391	0.052	0.012
BHMS217	116	3	2.93	0.00	0.603	0.546	0.177	-0.102
BHMS429	116	2	1.90	0.00	0.078	0.090	0.223	0.145
BHMS328	116	6	3.45	0.36	0.379	0.417	**0.003**	0.094
BHMS230	116	13	8.62	0.94	0.741	0.757	0.183	0.025
BHMS176	116	5	3.46	0.45	0.284	0.349	**0.041**	0.189
BHMS304-1	116	10	5.47	0.05	0.457	0.493	0.000	0.078
SSsp2216	116	13	7.66	0.56	0.793	0.783	0.071	-0.009
SsaD157	116	14	9.92	0.00	0.828	0.811	0.648	-0.017
BHMS176A	116	2	2.00	0.00	0.310	0.357	0.189	0.135
*Wild1*								
Ssa197	33	9	8.60	0.08	0.758	0.817	**0.024**	0.088
BHMS259	33	4	3.63	0.00	0.394	0.378	0.728	-0.026
BHMS217	33	3	3.00	0.00	0.545	0.512	0.718	-0.050
BHMS429	33	3	2.62	0.00	0.152	0.142	1.000	-0.053
BHMS328	33	4	3.51	0.00	0.333	0.369	0.170	0.111
BHMS230	33	8	7.18	0.74	0.667	0.786	**0.021**	0.166
BHMS176	33	4	3.27	0.00	0.212	0.195	1.000	-0.074
BHMS304-1	33	5	4.74	0.47	0.303	0.462	**0.000**	0.358
SSsp2216	33	7	6.38	0.00	0.758	0.782	0.516	0.047
SsaD157	33	19	16.02	0.95	0.848	0.918	0.083	0.091
BHMS176A	33	2	2.00	0.00	0.242	0.213	1.000	-0.123
*Wild2*								
Ssa197	61	8	7.26	0.00	0.803	0.812	0.315	0.019
BHMS259	61	5	3.92	0.29	0.230	0.253	0.356	0.100
BHMS217	61	2	1.34	0.00	0.016	0.016	NA	0.000
BHMS429	61	3	2.34	0.38	0.295	0.319	0.543	0.084
BHMS328	60	3	2.73	0.00	0.367	0.377	0.753	0.037
BHMS230	61	9	7.34	1.40	0.738	0.738	**0.007**	0.008
BHMS176	61	3	2.34	0.20	0.180	0.192	0.516	0.069
BHMS304-1	61	10	6.57	1.64	0.721	0.722	0.219	0.009
SSsp2216	60	6	5.41	0.58	0.700	0.770	0.089	0.099
SsaD157	61	21	14.32	1.80	0.820	0.886	0.145	0.083
BHMS176A	61	2	1.57	0.00	0.000	0.032	**0.008**	1.000

#### Mitochondrial DNA

To estimate the genetic diversity in populations, we calculated the number of haplotypes (*H*), nucleotide diversity (π) and haplotype diversity (*h*), based on the observed number of segregating sites using DnaSP v. 5.01 [48]. Relationships among haplotypes were analyzed with a haplotype network obtained by the median-joining method [49] using NETWORK ver. 4.6.1.1. (Fluxus Technology Ltd.). Population divergence estimates were obtained as *θ*_ST_ values in ARLEQUIN v3.5.1.2 [50] based on haplotype frequencies and pairwise differences among haplotypes. We used the Tajima and Nei [51] method of distance calculation. The significance of pairwise *θ*_ST_ values was ascertained by 1,000 permutations.

We also attempted to make inferences about the demographic history of the species and populations studied. First, we compared nucleotide and haplotype diversity. In populations that had undergone recent demographic growth, high haplotype diversity in conjunction with low nucleotide diversity should be observed, whereas high haplotype and nucleotide diversity would suggest a longstanding population [52]. In contrast, low values for both indicators are expected where a particular region of the genome is strongly affected by selection. Next, the expansion coefficient (*S/d*) was calculated to assess differences between recent and historical population sizes. If the ratio of number of variable sequence positions (*S*) to mean number of pairwise nucleotide differences among haplotypes (*d*) is large, this could indicate a recent population expansion, whereas low values for the ratio would suggest constant long-term population size [53].

The historical patterns affecting the present distribution of genetic variability were also investigated using the mismatch distribution of pairwise nucleotide differences. If a population has undergone rapid expansion, a unimodal mismatch distribution, approximating a Poisson curve, is to be expected [54], whereas populations approaching mutation drift equilibrium produce a multimodal (‘ragged’) mismatch distribution. Hence, we estimated the raggedness index (*r*) and assessed its statistical significance [55].

We also employed a range of neutrality tests to detect traces of past population growth or stability via mtDNA sequences [56]. Fu’s *F*_S_ test statistic [57] uses information from the haplotype distribution to test specifically for population growth, and it has been shown to be among the best statistics for detecting population growth in comparisons of statistical power [56, 57]. Low *F*_S_ values indicate an excess of single substitutions usually due to expansion.

Finally, we used Tajima’s *D*-test [58], which contrasts the number of nucleotide differences between sequences and the number of differences between segregating sites. Population expansions should cause significant negative departures of Tajima’s *D* from 0 [58]. Fu and Li’s D* and F* statistics [59] were calculated for comparison with Fu’s *F*_S_. The effects of background selection can be distinguished from population growth or range expansion by examining the pattern of significance between *F*_S_, F*, and D*. If *F*_S_ is significant and F* and D* are not, then population growth or range expansion is indicated, whereas the reverse pattern suggests selection [57]. All of these calculations were completed using DnaSP and ARLEQUIN.

### Ethics statement

Fish for this study were supplied by three fish farms breeding the Sevan trout in Armenia and a governmental institution (‘Sevan’ National Park of Ministry of Nature Protection of Armenia) executing scientific catches at a regular basis. Fish sampling in Lake Sevan was granted by the Ministry of Nature Protection in Armenia. No permission was required to capture the Sevan trout in Kirghizstan. In both wild and farm populations, fish were caught alive and, after ichthyologic study, a small portion of fat fin was taken for genetic analysis. Afterwards, all fish were returned to suppliers. During all procedures all efforts were made to minimize animals suffering. The study was approved by the Museum and Institute of Zoology, Polish Academy of Sciences.

## Results

### Microsatellites

We successfully amplified 11 microsatellite loci in all samples. The presence of null alleles was suggested by MICROCHECKER for the loci BHMS176 in *Farm3*, BHMS304-1 in *Wild1* and BHMS176A in *Wild2*. ML-NullFreq indicated that the frequency of ‘null alleles’ at these loci did not exceed 0.05. The fact that we amplified all loci in all individuals and that the presence of null alleles was indicated for different loci in different populations might suggest that the observation is interlinked with a population-specific distribution of alleles rather than a loci-specific problem, such as null alleles. Hence, we assumed that ‘null alleles’ had a minor influence on our results. These analyses did not reveal any large allele drop-out or genotypic errors either. We found one significant linkage disequilibrium between the Ssa197 and BHMS176 loci, but only in one population—*Wild1*. The usefulness of these loci in studies of population genetics of the species was determined using the polymorphic information content (PIC) for each locus (Table 1). Values estimated for the majority of loci were moderate, ranging from 0.16 to 0.41. At four loci the PIC index assumed a high value (in the range 0.74–0.91). For a total dataset (all individuals analysed as a single group), several loci deviated from Hardy-Weinberg equilibrium (HWE) (Table 1), due to heterozygote deficiency as indicated by *F*_IS_ values higher than zero.

At the population level, only *Farm2* was in HWE after FDR correction. In each of the examined populations none of the loci deviated from the HWE (Table 2); also none of the *F*_IS_ values proved significant following FDR correction. All indices of genetic variability were the highest in *Farm1* (Tables 2 and 3), except for *H*_O_, which reached its highest value in *Farm2*. The *F*_ST_ indicated limited genetic differentiation among the studied groups (Table 4). Only for comparisons with population *Wild2* we detected a moderate value for *F*_ST_.

**Table 3:** Mean genetic variability indices for 11 microsatellite loci of Sevan trout from four populations and groups of populations. *A*—number of alleles; *R*—allelic richness; *P*_A_—private alleles; *R*_P_*—*private allelic richness; *H*_O_—heterozygosity observed; *H*_E_—heterozygosity expected; *HWE*—*P-*values for HWE exact test for heterozygote deficiency/excess; *F*_IS_—fixation index (none of the values was significant after FDR correction), ^F^—significant after FDR correction.

Population	*A*	*R*	*P*_A_	*R*_P_	*H*o	*H*_E_	*HWE*	*F*_IS_
*Farm1*	7.54	7.31	1.18	1.65	0.538	0.580	0.0021^F^	0.095
*Farm2*	6.36	5.85	1.00	0.86	0.542	0.499	0.2663	-0.069
*Farm3*	7.45	5.11	0.64	0.23	0.506	0.521	0.0001^F^	0.033
*Wild1*	6.18	5.54	0.18	0.20	0.474	0.507	0.0041^F^	0.088
*Wild2*	6.54	5.01	0.82	0.57	0.443	0.465	0.0069^F^	0.057

**Table 4:** Among-localities genetic differentiation based on pairwise *F*_ST_ sensu Weir and Cockerham [33]. All *F*_ST_ values are significant after Bonferroni correction (Bonferroni corrected *P*-value, based on 200 permutation, at *α* = 0.05 was 0.005).

Population	*Farm2*	*Farm3*	*Wild1*	*Wild2*
*Farm1*	0.033	0.044	0.023	0.071
*Farm2*		0.051	0.025	0.078
*Farm3*			0.030	0.099
*Wild1*				0.050

**Fig 2:**
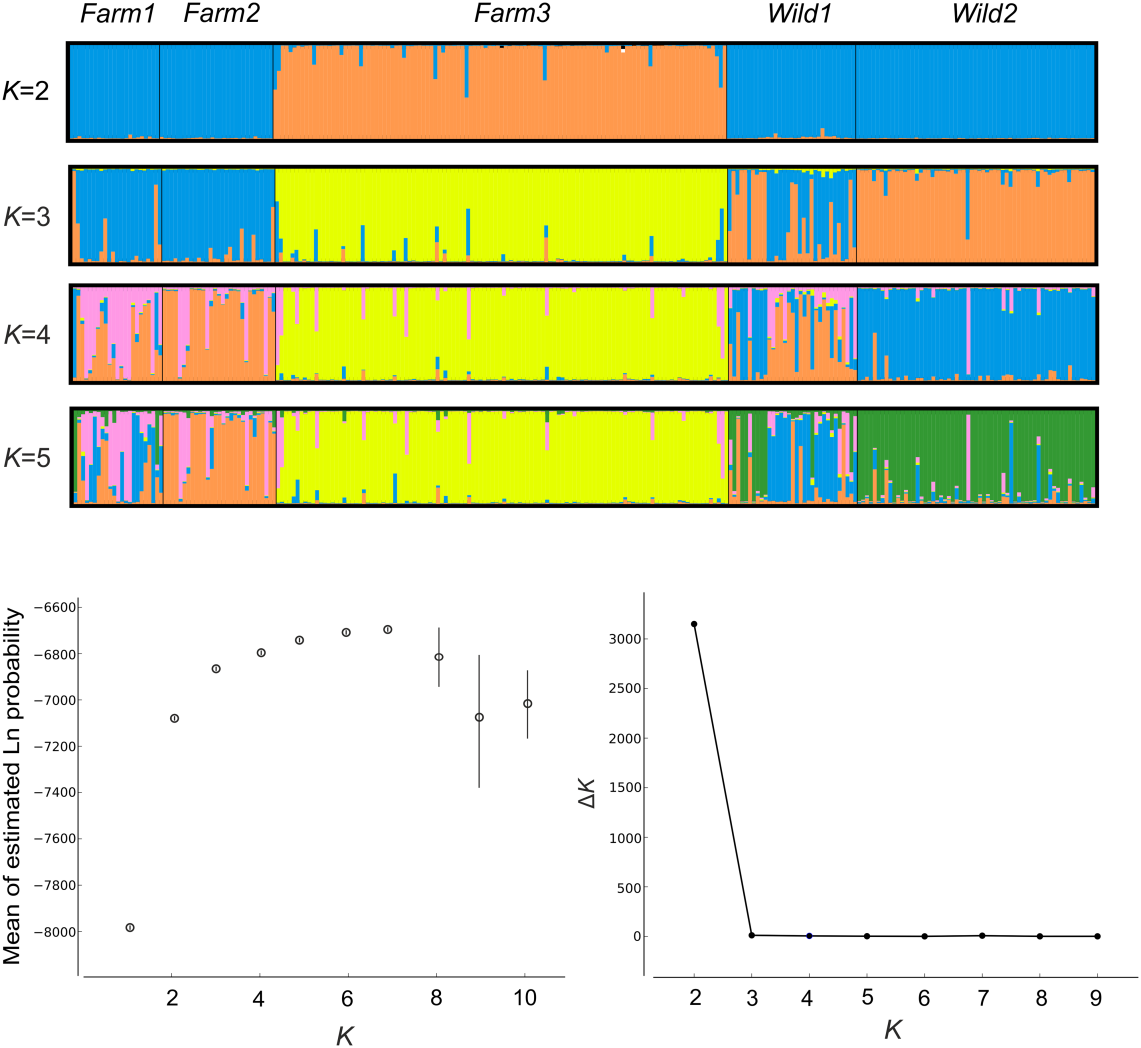
Bayesian assignment of individuals to genetic groups as suggested by STRUCTURE. The optimal number of clusters, indicated by Δ*K* analysis was 2. Bar plots for *K* = 2–5 (above), estimated mean likelihoods of each number of genetic clusters (below left) and Δ*K* curves as a function of *K* (below right) are presented.

Analysis in STRUCTURE (Fig 2) suggested the presence of two (Δ*K*) genetic groups. For this scenario, all individuals from *Farm3* were separated from other populations (Fig 2A, orange bars), forming a homogenous cluster (blue bars). In a five-group scenario (the number of genetic groups is equal to the number of studied populations) *Farm3* was similarly separated from other populations (yellow bars) but also *Wild2* constituted a distinct cluster (green bars), however with some signs of admixture with farm stocks (lilac bars) and *Wild1* (blue bars). Also, individuals assigned to the ‘green’ cluster were found in the *Wild1* population. *Farm1*, *Farm2* and *Wild1* showed signs of admixture. For higher values of *K*, particular individuals showed high levels of admixture and were distributed among clusters in a pattern that is difficult to explain, considering the watersheds organization and the history of stocking.

Migration rates calculated using BayesAss were low for all comparisons among populations (Table 5), suggesting the present isolation. The highest migration rate (0.203) was found between *Farm1* and *Farm2*, indicating that fish from *Farm2* had probably been introduced into the breeding stock of *Farm1*. In *Wild1*, we found a small fraction of individuals (0.12–0.16) derived from *Farm3* and *Wild2*. However, in the latter case this result reflects rather historical relationships between wild populations, as individuals from *Wild2* had never been reintroduced to *Wild1*.

**Table 5:** Matrix of inferred migration rates (SD) in BayesAss—values show the fraction of individuals in the population indicated in column 1, derived from populations from subsequent columns. On the diagonal (in bold)—the proportion of ‘residents’ in a particular population.

Population	*Farm1*	*Farm2*	*Farm3*	*Wild1*	*Wild2*
*Farm1*	**0.699 (0.201)**	0.203 (0.052)	0.047 (0.037)	0.015 (0.014)	0.036 (0.026)
*Farm2*	0.010 (0.010)	**0.945 (0.026)**	0.019 (0.017)	0.010 (0.010)	0.015 (0.014)
*Farm3*	0.009 (0.005)	0.012 (0.007)	**0.965 (0.011)**	0.004 (0.004)	0.010 (0.071)
*Wild1*	0.007 (0.007)	0.027 (0.017)	0.167 (0.032)	**0.676 (0.009)**	0.122 (0.031)
*Wild2*	0.005 (0.005)	0.007 (0.006)	0.007 (0.006)	0.005 (0.005)	**0.976 (0.011)**

None of the sample sites showed evidence of heterozygosity excess under the two-phase mutation model of microsatellite evolution, providing no genetic evidence for a recent bottleneck. In agreement with this finding, the distribution of allele frequency was normal (L-shaped), suggesting stability of effective population size. On the other hand, Garza-Williamson’s *M*-values were below critical values for most assumed estimates of *N*_e_ (Table 6), showing a bottleneck signature in the past.

**Table 6:** Results of bottleneck analysis, based on heterozygosity excess test, allele frequency analysis and Garza and Williamson’s *M-*ratio. *M*_C_—critical value of *M* for three levels of *θ*.

Population	Average heterozygosity excess	*P*-value	Allele frequency	Average *M*-ratio observed	*M*_C_			*P*-value
					*θ* = 0.2	*θ* = 1	*θ* = 2	
*Farm1*	-0.448	0.861	L-shaped	0.744	0.837	0.779	0.742	0.049
*Farm2*	-0.832	0.958	L-shaped	0.707	0.841	0.783	0.745	0.012
*Farm3*	-0.722	0.992	L-shaped	0.701	0.802	0.789	0.711	0.071
*Wild1*	-0.354	0.767	L-shaped	0.756	0.838	0.785	0.752	0.056
*Wild2*	-0.653	0.973	L-shaped	0.715	0.841	0.788	0.755	0.011

**Fig 3:**
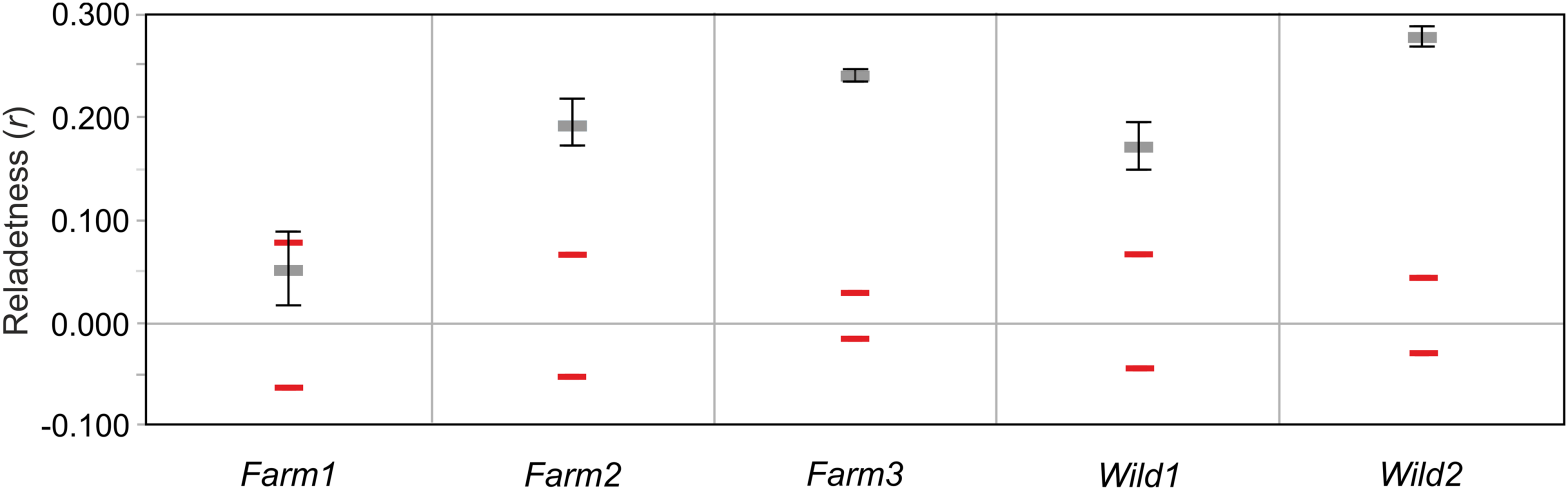
Graphic representation of the relatedness analysis. Red lines encompass 95% confidence interval (CI) about the null hypothesis of ‘No Difference’ across the populations as determined by permutation procedure. Whiskers around the mean indicate 95% CI of the mean values as determined by bootstrap resampling.

The average relatedness of hatchery fish ranged from 0.053 (*Farm1*) to 0.240 (*Farm3*) (Fig 3). In *Wild1* the relatedness was lower than in *Wild2* (0.172 vs. 0.278). In line with this finding, *Wild2* also had the lowest heterozygosities observed and expected.

Application of the linkage disequilibrium (LD) method [46] indicated the effective population size *N*_e_ = 110.9 [95% CI = 72.5–208.2] for *Wild2* when all alleles were used, and infinity [95% confidence interval (CI) = 297.6–∞], when alleles with frequencies below 0.05 were removed from the analysis. In *Wild1*, the relevant figures were: *N*_e_ = 14.4 [10.8–19.4] and *N*_e_ = 41.7 [31.1–59.3] for 0.05-allele frequency exclusion and no allele frequency exclusion levels, respectively. In farm populations, these estimates were at low levels similar to those noted for *Wild1*. The effective population size was *N*_e_ = 30.0 [16.7–79.5] and 39.4 [26.2–70.4] for *Farm1*, and *N*_e_ = 29.6 [27.9–31.5] and 38.7 [29.2–58.4] for *Farm3*, with lowest values for *Farm2* (*N*_e_ = 17.9 [12.4–27.7] and 22.9 [17.0–32.5]). ONESAMP indicated a large effective population size in *Wild2*, relative to other studied populations (*N*_e_ = 2,523.5 [1,149.8–3,3216.9]), and low in *Wild1* (*N*_e_ = 83.2 [56.7–226.6]), with slightly higher levels in *Farm1* and *Farm3* (respectively: *N*_*e*_ = 109.0 [73.5–335.4] and *N*_e_ = 131.1 [125.7–142.6]) and the lowest in *Farm2* (*N*_e_ = 72.9 [55.3–146.7]).

### Mitochondrial DNA

**Fig 4:**
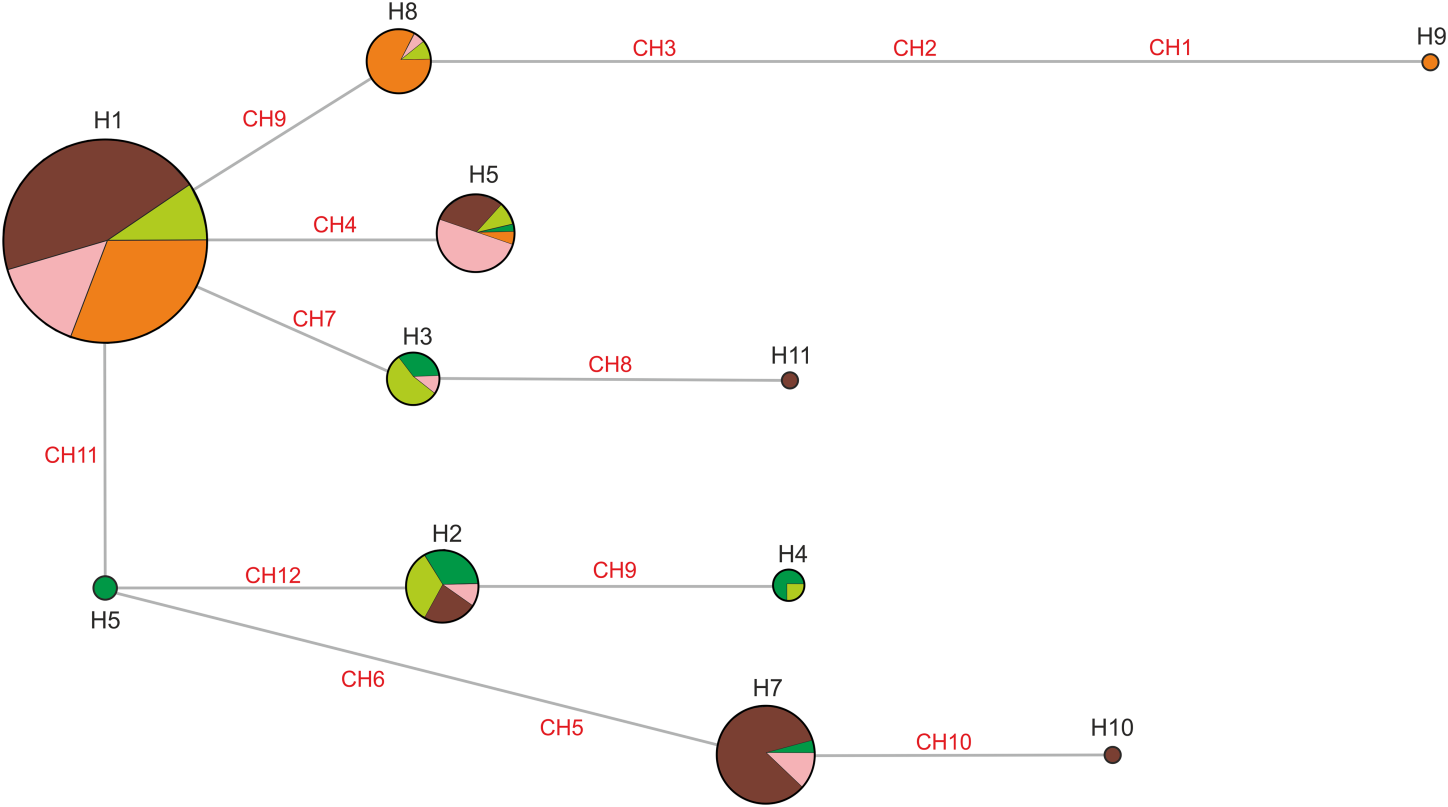
Median-joining network of haplotypes. The size of a circle is proportional to the frequency of a haplotype in the total sample. The pie charts correspond to the frequency of the given haplotype in particular populations: *Farm1*—dark green; *Farm2*—light green; *Farm3*—brown; *Wild1*—pink; *Wild2*—orange. CH1–CH12 indicate nucleotide substitutions, differing haplotypes.

We successfully amplified and sequenced a 905 bp fragment of the control region from 268 samples of the Sevan trout. Among obtained sequences we identified 11 haplotypes, defined by nine transitions and three transversions. Median-joining network (Fig 4) suggested that H1 was the core haplotype, being the most frequent in all populations except of *Farm1* (Table 7). In general, haplotypes differed by single substitutions (Fig 4; mean number of nucleotide differences among haplotypes = 1.438202 ± 0.879219), except of H9, found only in *Wild2*. The highest haplotype diversity was discovered in *Farm1*, the lowest in *Wild2* (Table 7). Nucleotide diversity was similar in populations studied, although clearly lower in *Wild2*.

**Table 7:** Genetic diversity indices estimated based on polymorphism of mitochondrial DNA and frequency of haplotypes. *n—*number of samples successfully analyzed; H—number of haplotypes identified; *h*—haplotype diversity; π —nucleotide diversity. Standard deviation shown in parentheses.

Population	*n*	H	*h*	π	Haplotype										
					H1	H2	H3	H4	H5	H6	H7	H8	H9	H10	H11
*Farm1*	21	6	0.824 (0.048)	0.0019 (0.0001)	0.19	0.33	0.19	0.14	0.10	0.05	0	0	0	0	0
*Farm2*	29	6	0.771 (0.047)	0.0019 (0.0002)	0.38	0.24	0.21	0.04	0	0.07	0.07	0	0	0	0
*Farm3*	113	6	0.566 (0.040)	0.0016 (0.0001)	0.60	0.04	0	0	0	0.07	0.26	0.02	0	0	0.01
*Wild1*	43	7	0.651 (0.061)	0.0015 (0.0003)	0.54	0.05	0.02	0	0	0.26	0.09	0.02	0	0.02	0
*Wild2*	62	4	0.405 (0.061)	0.0005 (0.0001)	0.74	0	0	0	0	0.02	0	0.23	0.02	0	0
Overall	268	11	0.644	0.0016 (0.0001)	0.567	0.078	0.041	0.015	0.007	0.082	0.134	0.063	0.004	0.004	0.004

We did not find significant genetic differentiation between *Farm1* and *Farm2* (Table 8). The highest *θ*_ST_ was noted for comparison of *Wild2* with farm populations, as well as between *Farm1* and *Farm3*. Comparison of *Farm2*, *Farm*3 and *Wild1* indicated low values of pairwise *θ*_ST_, suggesting small genetic differentiation. In general, *θ*_ST_ calculated from haplotype frequencies and genetic distance among haplotypes indicated very similar pattern of differentiation (Table 8).

**Table 8:** Genetic differentiation among studied populations estimated using *θ*_ST_ based on (i) distances among haplotypes (Tajima and Nei method—above diagonal) and (ii) based on frequency of haplotypes.

Population	*Farm1*	*Farm2*	*Farm 3*	*Wild1*	*Wild2*
*Farm1*		0.0291	0.2153*	0.2426*	0.3796*
*Farm2*	0.0033		0.0922*	0.0765*	0.1827*
*Farm 3*	0.2270*	0.1067*		0.0908*	0.2071*
*Wild1*	0.1560*	0.0626*	0.0439*		0.1009*
*Wild2*	0.3302*	0.2038*	0.1094*	0.1168*	

*—significant permutation test for genetic differentiation (*P* < 0.05).

The expansion coefficient (*S*/*d*) assumed highest values for *Wild2*, suggesting a recent expansion of this population. None of Tajima’s *D* and Fu’s *F* appeared to be significant, although the values were negative in majority of populations, suggesting recent expansion (Table 9). However, in *Wild2* we found evidence for background selection—F* and D* were negative and significantly different from zero. In all populations the raggedness index was low and non-significant.

**Table 9:** Results of neutrality tests. *S/d* ― expansion coefficient; *r* ― raggedness index significant values (*P* < 0.05) marked in bold.

Population	*S/d*	Tajima’s *D*	Fu’s *F*	Fu & Li F*	Fu & Li D*	*r*
*Farm1*	2.92	0.704	-0.616	0.537	0.372	0.049
*Farm2*	4.14	-0.155	-0.157	0.423	0.584	0.041
*Farm3*	5.36	-0.028	1.066	-0.316	-0.391	0.296
*Wild1*	6.06	-0.804	-1.241	-0.912	-0.765	0.079
*Wild2*	9.96	-1.226	-0.679	**-2.906**	**-2.784**	0.182

## Discussion

### Genetic variability and effective population size

Farm populations usually show a lower level of genetic variability than natural populations (e.g., [60, 61]). No such pattern was evident in our results, however, with farmed stock displaying a rather high level of genetic variability in comparison with both wild populations. On the other hand, there could be substantial variation in the level of genetic variability from one farm population to another. There is, for example an indication that breeding strains of brown trout of Atlantic origin in France show greater genetic variability than Mediterranean strains [62]. We noted that farm populations had higher heterozygosity than populations living in the wild. Moreover, the analysed wild populations of the Sevan trout seemed to have lower heterozygosity (as estimated using the comparable number of microsatellites) than wild populations of the other trout species, *Salmo trutta* and *S*. *salar* [63–66]. *Farm1* especially seems genetically diverse. One of the probable causes could be the forming of a stock based on a diverse gene pool, followed by maintenance of genetic diversity through the continued introduction of wild fish from Lake Sevan (*Wild1*). Indeed, the level of genetic differentiation estimated among these populations using microsatellites and a fragment of the mitochondrial genome is rather low. It is well known that even a low level of gene flow can prevent or at least retard the decrease of genetic variability in small populations (classical ‘one-migrant-per-generation’ rule—see [67, 68]). The farmed stock was established on the basis of individuals from the same *Wild1* population, and based on recent migration rates it is clear that fish from *Farm2* have already been released into the wild. Moreover, although genetic variability is usually lower in captive than wild populations and captivity may cause some undesirable physiological, behavioural and ecological changes, the fecundity and survival rates are often higher in captive than in wild populations. In salmonids, for example, survival from egg to smolt stages is typically 85–95% in hatcheries but only 1–5% in the wild [69]. Hence, we can assume that high productivity of farm stocks allowed to retained genetic variability of these fish and erased differences between farm and wild populations. Alternatively, it may be that we failed to detect significant genetic differences because genetic variability has already decreased in wild populations. Low genetic diversity in the Kirghiz sample seems to be easy to explain, as introduced populations often suffer genetic diversity decline following sequential founder events (e.g., [70–72]). The documented history indicates *S*. *ischchan* was introduced to Kirghizstan from Lake Sevan approximately 80 years ago, in 1930 and 1936 [10], and has been isolated since the introductions. This isolation was confirmed by the results of genetic differentiation analysis. Hence, genetic drift should eliminate substantial portion of neutral genetic variation. Our survey revealed no clear loss of alleles for the introduced Kirghiz (*Wild2*) population as compared to the source population from Lake Sevan, and the Kirghiz population actually shows the increased number of private alleles. However, as the Kirghiz population has not been genetically reinforced from other sources than Lake Sevan, we could assume that the difference reflects rare allele loss in the native population. Accordingly, in the Kirghiz population we also found a unique haplotype (H9), absent in Lake Sevan. We also observed relatively little reduction (7.5%) in the allelic richness (*R*) of introduced populations relative to the native population. Moreover, microsatellite allelic diversity in the Kirghiz population seems to be at a level comparable with that estimated for other trout species [63, 65]. Differences in a level of genetic diversity are clearer in the case of mitochondrial DNA. The effective population size of mitochondrial markers is lower than bi-parentally inherited microsatellites, hence the founder effect is more pronounced in the case of mtDNA.

### Genetic effects of the past process

Based on the results of neutrality tests, we found evidence that introduction to Lake Issyk Kul had caused expansion of the Kirghiz population (*Wild2*), probably due to availability of much larger habitat, as compared to farm populations, and more stable habitat, than in the case of the Sevan population (*Wild1*), where severe habitat perturbations had occurred. Interestingly, despite the largest effective population size, the Kirghiz population manifested the highest average relatedness. Although non-random sampling cannot be excluded entirely, we suggest that the observed pattern had been shaped by expansion of a limited gene pool following introduction. The overall size of the Sevan population has most probably been decreasing, whereas the Sevan trout from Kirghizstan has maintained a large population. Indeed, our analysis clearly suggests that the effective population size observed presently is much higher in the introduced Kirghiz population than in a source population from Lake Sevan, and it is known, for example, that population specific differences in the effective population size may alter genetic diversity [73]. The hypothesis concerning decrease of genetic diversity in the native population is consistent with past water management of the lake. Lake Sevan went through highly manipulated water-level regulations, continued since 1940 when the waters began to be used for power generation and irrigation. The lowest water level was reached in 1952. This factor had a very negative impact on the natural propagation of the trout, and a majority of the spawning grounds were drained [74]. Poaching also impacted dramatically upon the condition of the fish stock in the lake. Currently most of the fish are propagated by hatcheries, and the species was declared protected (since 1989 it has been included in the Red Data Book of Armenia). On the other hand, a similar situation has been observed in Kirghizstan. By 1960, the Sevan trout had become rare in Issyk Kul, due to a shortage of suitable breeding areas, and seed for stocking the lake was produced by two hatcheries [75]. However, this population probably rebounded faster demographically, given that in new environment, the biology and morphology of Sevan trout in Lake Issyk Kul have considerably changed, probably due to phenotypic plasticity (see, e.g. [76]). The fecundity has increased 5–6 times (the average production is 7,460 eggs versus 1,376 eggs before the water decline in the native gegarkuni form [73]), and the Issyk Kul trout mature one to two years earlier than those of Sevan Lake, and males attain sexual maturity at the age of three years (2+), females at five years (4+) [17]. The Sevan gegarkuni usually mature at the age of six years [77] although they could also mature at the age of 3+ (females) or 2+ (males) [78], as recently happens in farms (A.N. Asatryan, unpublished data). Fish from Issyk Kul are also much bigger, reaching 89 cm in body length and 17 kg in body mass, while in Lake Sevan they rarely attain 60 cm and 4 kg ([75]; cf. [17]).

In contrast to the Kirgiz population, individuals from *Farm1* were rather distantly related. Knowing that this hatchery has been used to ensure the survival of large numbers of fry, and the broodstock is continually taken from the wild population in Lake Sevan, increase of relatedness and inbreeding depression is unlikely to occur [79].

Several lines of evidence suggest that populations analyzed in this study experienced a bottleneck event pre-dating the present study despite the fact that the bottleneck was not detectable using either tests of excess heterozygosity under the TPM or mode-shift indicators as qualitative descriptors of allele frequency distribution. However, it is well known that there is less statistical power to detect a bottleneck with these tests, unless a strong bottleneck is very recent and/or ongoing, because heterozygosity rapidly returns to equilibrium values [80]. On the other hand, the test based on the total number of alleles and the range of allele sizes provided *M*-ratios consistently below critical thresholds of a sample drawn from a small (*θ* = 0.2) to moderate (*θ* = 1.0) population and at the margin of significance in a large population (*θ* = 2.0), indicating a bottleneck and suggesting reduction in population size.

Summarizing our findings, we can state that either (i) human induced gene flow among *Farm1*, *Farm2*, *Farm 3*, and *Wild1* prevents the decrease of genetic variability in hatcheries or (ii) a low level of genetic variability in farm populations, alongside a decreased level of genetic variability in wild populations, results in an inability to detect differences. However, in this case the processes causing this would be different. In farm populations low levels of genetic diversity are likely due to founder effect and low effective population size. In *Wild1*—the decrease in the number of individuals (and subsequent elimination of unique/low frequency alleles, resulting in a low number of private alleles), as well as a low effective population size, and the existence of internal sub-structure, are the likely causes of low genetic variation. Nevertheless, further studies are required to confirm this last hypothesis. In *Wild2—*a founder effect following an introduction based on a large, but obviously nonetheless limited number of founders, as well as present isolation (*F*_ST_), high relatedness coefficient and the lowest heterozygosity are the likely explanations for why this population is genetically depauperate. Additionally, (iii) there is ongoing admixture of genetic material between farm populations and *Wild1* (*F*_ST_, STRUCTURE, BayessAss), whereas *Wild2* is isolated from other investigated populations. Genetic differentiation and observed phenotypic changes in fish from *Wild2* suggest that natural selection may also play some role in the genetic identity of this population. Further studies, using methods allowing neutral and adaptive genetic variation to be distinguished are required to indicate which factor has had the stronger effect.

One of the most important parameters affecting genetic diversity is effective population size (*N*_e_) [81]. In farms and Lake Sevan, *N*_e_ was clearly lower than in the naturalized population. This is another indication, that population size in native region has been decreasing. Nevertheless, all methods of computing *N*_e_ include underlying assumptions (e.g., [46]) that are rarely fulfilled in the populations in which they are implemented and these estimations should be treated with some caution. However, they provide indications regarding each population’s effective size, and thus potential for genetic drift and inbreeding.

### Genetic differentiation

In terms of further species management, it would be useful to estimate if the level of differentiation of the wild Kirghiz population from the farm stock and the source population from Lake Sevan (which seems rather small for neutral microsatellites), should be interpreted as great enough to rule out possible translocations. Female Chinook salmon (*Oncorhynchus tshawytscha*) from a hatchery were, for example, found to have smaller eggs and reduced reproductive success relative to the wild populations [82], and farm populations might be ‘sub-optimal’ for translocations. Heath et al. [82] also suggest that genetic adaptation in fish occurs very rapidly, and indeed the Kirghiz population has already gained some important biological and morphological features not observed in the source population. Nonetheless, at this point, we can still assume that the level of differentiation is rather low. However, analyses based on neutral markers usually screen a small proportion of the genome, affected primary by drift and mutations, whereas functional genes, involved in adaptations, are additionally affected by selection (e.g., [83, 84]). Hence, we strongly recommend to minimize the handling of the only native population of the study (Lake Sevan) and in particular not to introduce the derived stocks from farms or the Lake Issyk Kul trout. Future relocations of Sevan trout into and eventually from the Kirghiz population (where these fish are treated as a potential pest [85]) should be preceded be additional genetic analyses, having in mind that *S*. *ischchan* is closely related to *S*. *trutta*, one of the most invasive species of the world, according to IUCN [86]. For example, genome scans could be used to identified ‘outlier’ loci that may be under divergent selection (e.g., [87–89]). This would allow for an estimation of contemporary genetic differentiation, as shaped by selection and adaptive processes—in a strategy used more and more frequently in the conservation and management of wild populations [90–92]. An introduction of already ‘genetically incompatible’ individuals could be unsuccessful, as they will lose out in competition with a better-adapted, resident population or, even more importantly, might even prove harmful to the resident population thanks to outbreeding depression. Indeed, this phenomenon has already been reported as an important factor reducing management efforts in wild populations [93], including fish species [94, 95].

## Supporting information

S1 Table Two-digit genotypes of individuals of *S*. *ischchan* at 11 microsatellite loci. (DOC)
