# Supplementary material for: Fish introductions in the former Soviet Union: The Sevan trout (Salmo ischchan) — 80 years later
Source: PLoS One. 2017 Jul 6;12(7):e0180605. doi: 10.1371/journal.pone.0180605 (PMC5500335; doi:10.1371/journal.pone.0180605)
Supplement: S1 Table — (DOC) [file pone.0180605.s001.doc]

S1 Table. Two-digit genotypes of individuals of *S. ischchan* at 11 microsatellite loci.

| Ssa197 | BHMS259 | BHMS217 | BHMS429 | BHMS328 | BHMS230 | BHMS176 | BHMS304-1 | SSsp2216 | SsaD157 | BHMS176A |
| --- | --- | --- | --- | --- | --- | --- | --- | --- | --- | --- |
| *Population: Wild1* | | | | | | | | | | |
| 0709 | 0404 | 0606 | 0404 | 0404 | 0505 | 0404 | 0505 | 1212 | 1026 | 0101 |
| 0506 | 0404 | 0606 | 0404 | 0405 | 0508 | 0404 | 0507 | 0911 | 0520 | 0101 |
| 0106 | 0404 | 0606 | 0000 | 0405 | 0817 | 0404 | 0709 | 0911 | 0514 | 0101 |
| 0109 | 0104 | 0909 | 0000 | 0406 | 1319 | 0405 | 0505 | 0511 | 2426 | 0102 |
| 0606 | 0404 | 0607 | 0404 | 0405 | 0509 | 0404 | 0709 | 0511 | 0923 | 0102 |
| 0606 | 0404 | 0606 | 0404 | 0304 | 0509 | 0204 | 0509 | 0510 | 0715 | 0101 |
| 0607 | 0404 | 0609 | 0404 | 0304 | 0505 | 0404 | 0514 | 0910 | 0511 | 0101 |
| 0506 | 0404 | 0609 | 0404 | 0404 | 0505 | 0404 | 0505 | 1119 | 1018 | 0101 |
| 0206 | 0404 | 0606 | 0404 | 0404 | 0508 | 0404 | 0505 | 1011 | 0430 | 0101 |
| 0708 | 0304 | 0708 | 0404 | 0304 | 1414 | 0404 | 0507 | 0919 | 1222 | 0101 |
| 0407 | 0404 | 0707 | 0404 | 0505 | 0517 | 0404 | 0507 | 1112 | 2228 | 0102 |
| 0203 | 0102 | 0707 | 0404 | 0303 | 1717 | 0204 | 0101 | 1316 | 2424 | 0102 |
| 0607 | 0404 | 0607 | 0404 | 0304 | 0511 | 0404 | 0709 | 0912 | 1527 | 0202 |
| 0610 | 0104 | 0606 | 0404 | 0304 | 0517 | 0505 | 0914 | 1118 | 0925 | 0101 |
| 0408 | 0104 | 0609 | 0404 | 0303 | 0817 | 0205 | 1213 | 1720 | 0513 | 0202 |
| 0609 | 0406 | 0609 | 0404 | 0404 | 0105 | 0204 | 0505 | 0910 | 0710 | 0101 |
| 0909 | 0404 | 0606 | 0404 | 0405 | 0508 | 0205 | 0511 | 1011 | 0715 | 0101 |
| 0207 | 0404 | 0608 | 0404 | 0304 | 0505 | 0404 | 0511 | 0810 | 0714 | 0101 |
| 0608 | 0406 | 0709 | 0404 | 0506 | 0505 | 0404 | 0611 | 0911 | 1214 | 0101 |
| 0607 | 0404 | 0606 | 0404 | 0405 | 0508 | 0404 | 0711 | 0511 | 1212 | 0101 |
| 0609 | 0306 | 0607 | 0404 | 0304 | 0505 | 0204 | 0505 | 1015 | 0721 | 0202 |
| 0206 | 0404 | 0607 | 0404 | 0404 | 0508 | 0404 | 0505 | 0812 | 0929 | 0101 |
| 0507 | 0606 | 0606 | 0404 | 0404 | 0509 | 0404 | 0709 | 1112 | 3434 | 0101 |
| *Population: Wild2* | | | | | | | | | | |
| 0506 | 0404 | 0607 | 0404 | 0304 | 0508 | 0404 | 0505 | 0610 | 2022 | 0101 |
| 0506 | 0304 | 0606 | 0404 | 0304 | 0508 | 0404 | 0507 | 0609 | 1222 | 0101 |
| 0506 | 0406 | 0606 | 0404 | 0404 | 0811 | 0404 | 0505 | 0610 | 1024 | 0101 |
| 0207 | 0404 | 0707 | 0404 | 0406 | 0505 | 0404 | 0505 | 1111 | 2024 | 0101 |
| 0607 | 0104 | 0609 | 0404 | 0404 | 0505 | 0104 | 0305 | 0308 | 0330 | 0101 |
| 0507 | 0404 | 0609 | 0404 | 0404 | 0516 | 0404 | 0512 | 0208 | 1630 | 0101 |
| 0509 | 0404 | 0606 | 0204 | 0304 | 1111 | 0404 | 0509 | 1113 | 1822 | 0101 |
| 0507 | 0404 | 0607 | 0304 | 0404 | 0808 | 0404 | 0505 | 0611 | 2224 | 0101 |
| 0506 | 0404 | 0606 | 0304 | 0304 | 0808 | 0204 | 0507 | 0610 | 1024 | 0101 |
| 0506 | 0404 | 0606 | 0404 | 0404 | 0810 | 0404 | 0505 | 0913 | 0522 | 0101 |
| 0508 | 0404 | 0607 | 0404 | 0304 | 0808 | 0404 | 0505 | 0913 | 1222 | 0101 |
| 0507 | 0404 | 0709 | 0404 | 0404 | 1017 | 0405 | 0511 | 0208 | 1622 | 0102 |
| 0607 | 0406 | 0606 | 0404 | 0404 | 0508 | 0404 | 0709 | 1012 | 0532 | 0101 |
| 0508 | 0404 | 0606 | 0404 | 0404 | 0508 | 0404 | 0505 | 1213 | 0524 | 0101 |
| 0406 | 0405 | 0609 | 0304 | 0406 | 0404 | 0404 | 0505 | 1213 | 1016 | 0101 |
| 0506 | 0404 | 0609 | 0304 | 0304 | 0817 | 0404 | 0511 | 0607 | 1622 | 0101 |
| 0506 | 0304 | 0606 | 0404 | 0404 | 0810 | 0404 | 0505 | 1113 | 1225 | 0101 |
| 0206 | 0406 | 0606 | 0404 | 0406 | 0505 | 0404 | 0507 | 1011 | 1010 | 0101 |
| 0507 | 0405 | 0609 | 0404 | 0406 | 0510 | 0404 | 0505 | 0913 | 1024 | 0101 |
| 0207 | 0404 | 0607 | 0404 | 0406 | 0508 | 0204 | 0507 | 1011 | 2024 | 0101 |
| 0204 | 0304 | 0606 | 0404 | 0406 | 0508 | 0404 | 0505 | 1111 | 1233 | 0101 |
| 0506 | 0404 | 0607 | 0404 | 0404 | 0110 | 0404 | 0513 | 0506 | 2527 | 0101 |
| 0607 | 0406 | 0609 | 0404 | 0406 | 0508 | 0405 | 0711 | 0610 | 1016 | 0101 |
| 0206 | 0406 | 0606 | 0404 | 0404 | 0508 | 0404 | 0709 | 1112 | 0510 | 0101 |
| 0506 | 0406 | 0606 | 0404 | 0404 | 0508 | 0404 | 0506 | 0610 | 1022 | 0101 |
| 0405 | 0304 | 0607 | 0404 | 0404 | 0808 | 0404 | 0505 | 0611 | 1024 | 0101 |
| 0305 | 0404 | 0709 | 0404 | 0404 | 0517 | 0404 | 0305 | 0311 | 0322 | 0101 |
| 0506 | 0405 | 0707 | 0304 | 0404 | 0505 | 0405 | 0511 | 0812 | 2224 | 0101 |
| 0206 | 0404 | 0606 | 0404 | 0404 | 0404 | 0404 | 0509 | 0911 | 0916 | 0101 |
| *Population: Farm1* | | | | | | | | | | |
| 0308 | 0809 | 0103 | 0404 | 0304 | 0818 | 0405 | 0714 | 0616 | 1228 | 0202 |
| 0507 | 0809 | 0101 | 0505 | 0304 | 0514 | 0304 | 0909 | 1112 | 0620 | 0101 |
| 0505 | 0809 | 0304 | 0405 | 0405 | 0510 | 0404 | 0505 | 1010 | 1420 | 0202 |
| 0505 | 0808 | 0303 | 0404 | 0404 | 0507 | 0404 | 0505 | 0910 | 1818 | 0102 |
| 0107 | 0808 | 0303 | 0404 | 0404 | 0608 | 0404 | 0609 | 0910 | 0809 | 0101 |
| 0303 | 0910 | 0304 | 0404 | 0304 | 0515 | 0404 | 0509 | 0909 | 0710 | 0101 |
| 0205 | 0809 | 0303 | 0404 | 0505 | 0708 | 0404 | 0509 | 0610 | 1422 | 0101 |
| 0507 | 0910 | 0304 | 0405 | 0405 | 0508 | 0404 | 0509 | 0911 | 1014 | 0101 |
| 0505 | 0909 | 0303 | 0404 | 0404 | 0506 | 0202 | 0509 | 0910 | 1214 | 0101 |
| 0508 | 0809 | 0303 | 0404 | 0404 | 0107 | 0404 | 0505 | 1213 | 1820 | 0202 |
| 0306 | 0809 | 0101 | 0404 | 0404 | 0508 | 0204 | 0911 | 0511 | 1214 | 0101 |
| 0606 | 0809 | 0103 | 0404 | 0405 | 0505 | 0404 | 0505 | 0912 | 0714 | 0101 |
| 0506 | 0910 | 0303 | 0404 | 0405 | 0505 | 0404 | 0505 | 0912 | 1014 | 0102 |
| 0506 | 0808 | 0103 | 0404 | 0405 | 0506 | 0404 | 0509 | 1012 | 0918 | 0102 |
| 0207 | 0809 | 0303 | 0404 | 0404 | 0708 | 0204 | 0509 | 0610 | 1418 | 0101 |
| 0506 | 0809 | 0303 | 0404 | 0405 | 0505 | 0404 | 0509 | 1012 | 1420 | 0101 |
| 0608 | 0808 | 0304 | 0404 | 0404 | 0508 | 0204 | 0505 | 1012 | 1420 | 0102 |
| 0509 | 0809 | 0303 | 0404 | 0404 | 0507 | 0204 | 0507 | 1010 | 0614 | 0101 |
| 0205 | 0809 | 0303 | 0405 | 0405 | 0507 | 0404 | 0505 | 1010 | 1418 | 0101 |
| 0205 | 0809 | 0303 | 0404 | 0404 | 0507 | 0404 | 0505 | 1313 | 1418 | 0102 |
| 0105 | 0808 | 0104 | 0404 | 0405 | 0508 | 0204 | 0505 | 1010 | 0714 | 0101 |
| 0105 | 0809 | 0303 | 0404 | 0405 | 0107 | 0404 | 0505 | 1011 | 0914 | 0101 |
| 0506 | 0809 | 0303 | 0404 | 0305 | 0515 | 0404 | 0714 | 0911 | 1012 | 0101 |
| 0505 | 0909 | 0303 | 0404 | 0404 | 0815 | 0204 | 0509 | 0913 | 1414 | 0101 |
| 0506 | 0808 | 0303 | 0404 | 0404 | 0108 | 0404 | 0505 | 1012 | 0714 | 0202 |
| 0505 | 0809 | 0303 | 0404 | 0404 | 0505 | 0404 | 0509 | 0909 | 0714 | 0101 |
| 0505 | 0809 | 0303 | 0404 | 0404 | 0508 | 0202 | 0505 | 1010 | 1420 | 0101 |
| 0506 | 0808 | 0304 | 0404 | 0404 | 0808 | 0404 | 0505 | 0911 | 2020 | 0101 |
| 0206 | 0808 | 0303 | 0404 | 0404 | 0112 | 0404 | 0509 | 0913 | 0918 | 0102 |
| 0506 | 0810 | 0103 | 0404 | 0404 | 0105 | 0404 | 0508 | 0912 | 1020 | 0102 |
| 0303 | 0910 | 0304 | 0404 | 0304 | 0505 | 0404 | 0509 | 0909 | 0710 | 0101 |
| 0508 | 0809 | 0304 | 0404 | 0404 | 0505 | 0204 | 0508 | 0910 | 1420 | 0102 |
| 0608 | 0809 | 0303 | 0404 | 0404 | 0105 | 0204 | 0505 | 1012 | 1418 | 0102 |
| 0306 | 0809 | 0104 | 0404 | 0404 | 0505 | 0406 | 0613 | 1017 | 0709 | 0102 |
| 0508 | 0808 | 0103 | 0404 | 0404 | 0708 | 0405 | 0505 | 0910 | 0920 | 0101 |
| 0707 | 0909 | 0303 | 0404 | 0505 | 0812 | 0404 | 0505 | 1012 | 1414 | 0101 |
| 0606 | 0808 | 0304 | 0404 | 0405 | 0508 | 0204 | 0505 | 1012 | 0720 | 0101 |
| 0505 | 0909 | 0303 | 0404 | 0404 | 0507 | 0204 | 0509 | 1010 | 1414 | 0101 |
| 0105 | 0809 | 0303 | 0404 | 0405 | 0107 | 0404 | 0505 | 0911 | 0914 | 0101 |
| 0606 | 0809 | 0304 | 0404 | 0405 | 0505 | 0404 | 0505 | 1112 | 0714 | 0101 |
| 0105 | 0808 | 0103 | 0404 | 0405 | 0505 | 0404 | 0505 | 1012 | 0620 | 0101 |
| 0606 | 0809 | 0303 | 0404 | 0404 | 0508 | 0404 | 0710 | 0811 | 1020 | 0101 |
| 0508 | 0809 | 0303 | 0404 | 0404 | 0508 | 0204 | 0909 | 1011 | 1420 | 0101 |
| 0509 | 0809 | 0103 | 0404 | 0405 | 0505 | 0404 | 0507 | 1012 | 1418 | 0102 |
| 0505 | 0809 | 0304 | 0404 | 0404 | 0505 | 0204 | 0509 | 1012 | 1420 | 0101 |
| 0506 | 0909 | 0303 | 0404 | 0404 | 0506 | 0404 | 0505 | 1011 | 1414 | 0101 |
| 0606 | 0808 | 0303 | 0404 | 0404 | 0508 | 0404 | 0505 | 1213 | 1418 | 0102 |
| 0506 | 0909 | 0303 | 0404 | 0404 | 0707 | 0404 | 0505 | 1213 | 1414 | 0102 |
| 0105 | 0910 | 0303 | 0405 | 0405 | 0507 | 0404 | 0505 | 0912 | 1014 | 0102 |
| 0408 | 0808 | 0203 | 0404 | 0404 | 0508 | 0404 | 0509 | 0811 | 0824 | 0101 |
| 0105 | 0809 | 0303 | 0404 | 0404 | 0808 | 0406 | 0505 | 0910 | 1420 | 0101 |
| 0506 | 0809 | 0304 | 0404 | 0404 | 0508 | 0404 | 0505 | 1010 | 1014 | 0102 |
| 0508 | 0909 | 0304 | 0405 | 0404 | 0510 | 0204 | 0508 | 1010 | 1414 | 0101 |
| 0506 | 0808 | 0304 | 0404 | 0405 | 0505 | 0202 | 0505 | 1013 | 1420 | 0101 |
| 0505 | 0809 | 0103 | 0404 | 0404 | 0710 | 0404 | 0505 | 1011 | 0914 | 0101 |
| 0607 | 0809 | 0303 | 0404 | 0404 | 0105 | 0404 | 0505 | 1213 | 1014 | 0202 |
| 0206 | 0809 | 0303 | 0404 | 0404 | 0808 | 0404 | 0609 | 1012 | 1422 | 0101 |
| 0205 | 0808 | 0303 | 0404 | 0404 | 0505 | 0404 | 0505 | 1013 | 0718 | 0102 |
| 0607 | 0909 | 0305 | 0404 | 0405 | 0506 | 0404 | 0505 | 1012 | 1424 | 0102 |
| 0508 | 0909 | 0303 | 0404 | 0404 | 0510 | 0404 | 0509 | 1011 | 1414 | 0102 |
| 0508 | 0809 | 0304 | 0404 | 0404 | 0510 | 0206 | 0609 | 1011 | 1420 | 0101 |
| 0108 | 0809 | 0303 | 0405 | 0404 | 0708 | 0404 | 0505 | 0910 | 1420 | 0102 |
| 0205 | 0808 | 0303 | 0404 | 0404 | 0612 | 0204 | 0909 | 1012 | 1218 | 0101 |
| 0608 | 0808 | 0304 | 0404 | 0404 | 0505 | 0404 | 0505 | 1212 | 0909 | 0101 |
| 0505 | 0809 | 0303 | 0404 | 0405 | 0508 | 0404 | 0609 | 0910 | 1822 | 0101 |
| 0105 | 0808 | 0303 | 0404 | 0404 | 0505 | 0404 | 0505 | 1010 | 1818 | 0102 |
| 0506 | 0910 | 0303 | 0404 | 0404 | 0508 | 0404 | 0505 | 1212 | 1014 | 0101 |
| 0506 | 0809 | 0303 | 0404 | 0404 | 0506 | 0204 | 0609 | 0912 | 0922 | 0102 |
| 0506 | 0808 | 0303 | 0404 | 0404 | 0608 | 0204 | 0508 | 0611 | 1820 | 0101 |
| 0304 | 0809 | 0304 | 0404 | 0404 | 0808 | 0404 | 0510 | 1115 | 0918 | 0101 |
| 0106 | 0808 | 0101 | 0404 | 0404 | 0610 | 0404 | 0506 | 1011 | 0718 | 0102 |
| 0508 | 0809 | 0303 | 0404 | 0404 | 0508 | 0204 | 0609 | 0610 | 0714 | 0101 |
| 0505 | 0909 | 0304 | 0404 | 0505 | 0310 | 0404 | 0509 | 1212 | 1414 | 0101 |
| 0608 | 0808 | 0103 | 0404 | 0505 | 0310 | 0404 | 0506 | 0912 | 0920 | 0202 |
| 0508 | 0810 | 0303 | 0404 | 0506 | 0912 | 0404 | 0506 | 1012 | 1018 | 0102 |
| 0108 | 0809 | 0303 | 0405 | 0505 | 0910 | 0404 | 0506 | 0910 | 1420 | 0102 |
| 0206 | 0810 | 0303 | 0404 | 0505 | 0710 | 0404 | 0505 | 0404 | 0910 | 0102 |
| 0505 | 0809 | 0303 | 0404 | 0506 | 0809 | 0204 | 0506 | 1012 | 1418 | 0101 |
| 0505 | 0809 | 0304 | 0404 | 0505 | 0712 | 0404 | 0505 | 0910 | 0614 | 0101 |
| 0508 | 0909 | 0303 | 0404 | 0506 | 0710 | 0404 | 0506 | 0912 | 1414 | 0101 |
| 0506 | 0808 | 0303 | 0404 | 0404 | 0812 | 0204 | 0505 | 1011 | 1820 | 0101 |
| 0205 | 0909 | 0304 | 0404 | 0405 | 0508 | 0404 | 0505 | 0913 | 1414 | 0101 |
| 0608 | 0809 | 0303 | 0404 | 0404 | 0108 | 0404 | 0505 | 1012 | 1420 | 0101 |
| 0606 | 0809 | 0303 | 0404 | 0404 | 0508 | 0204 | 0509 | 0612 | 1414 | 0101 |
| 0505 | 0909 | 0304 | 0405 | 0405 | 0508 | 0404 | 0505 | 1012 | 1414 | 0101 |
| 0505 | 0809 | 0303 | 0404 | 0405 | 0508 | 0404 | 0505 | 0910 | 1822 | 0101 |
| 0507 | 0809 | 0304 | 0404 | 0405 | 0608 | 0404 | 0505 | 1011 | 1418 | 0101 |
| 0508 | 0809 | 0304 | 0404 | 0404 | 0508 | 0404 | 0509 | 1012 | 1014 | 0101 |
| 0606 | 1010 | 0103 | 0404 | 0404 | 0112 | 0204 | 0509 | 1013 | 1010 | 0101 |
| 0708 | 0809 | 0303 | 0404 | 0405 | 0505 | 0204 | 0508 | 1013 | 0518 | 0101 |
| 0108 | 0809 | 0303 | 0404 | 0404 | 0708 | 0404 | 0505 | 1011 | 0924 | 0102 |
| 0507 | 0909 | 0303 | 0404 | 0404 | 0810 | 0405 | 0505 | 1010 | 1414 | 0102 |
| 0506 | 0809 | 0303 | 0404 | 0405 | 0808 | 0404 | 0505 | 0612 | 0718 | 0101 |
| 0508 | 0809 | 0303 | 0404 | 0404 | 0508 | 0204 | 0909 | 0610 | 0714 | 0101 |
| 0506 | 0810 | 0303 | 0404 | 0405 | 0507 | 0404 | 0505 | 0910 | 0610 | 0101 |
| 0508 | 0809 | 0304 | 0405 | 0404 | 0510 | 0204 | 0505 | 1012 | 0614 | 0101 |
| 0406 | 0809 | 0304 | 0404 | 0405 | 0505 | 0304 | 0509 | 0511 | 1416 | 0102 |
| 0606 | 0808 | 0103 | 0404 | 0404 | 0808 | 0202 | 0509 | 1011 | 0914 | 0102 |
| 0505 | 0909 | 0303 | 0404 | 0405 | 0607 | 0404 | 0505 | 0912 | 1422 | 0101 |
| 0505 | 0809 | 0303 | 0404 | 0404 | 0512 | 0404 | 0509 | 0910 | 0714 | 0101 |
| 0505 | 0809 | 0303 | 0404 | 0404 | 0508 | 0202 | 0505 | 0610 | 1420 | 0101 |
| 0606 | 0809 | 0304 | 0404 | 0405 | 0505 | 0404 | 0505 | 1112 | 1420 | 0101 |
| 0106 | 0809 | 0304 | 0404 | 0404 | 0508 | 0404 | 0505 | 1010 | 1414 | 0202 |
| 0606 | 0809 | 0103 | 0404 | 0404 | 0808 | 0404 | 0505 | 0909 | 0724 | 0202 |
| 0506 | 0809 | 0304 | 0404 | 0405 | 0108 | 0305 | 0509 | 0911 | 1214 | 0102 |
| 0607 | 0809 | 0303 | 0404 | 0405 | 0505 | 0404 | 0505 | 0913 | 1822 | 0102 |
| 0506 | 0808 | 0303 | 0404 | 0405 | 0505 | 0404 | 0505 | 0613 | 1418 | 0102 |
| 0506 | 0909 | 0303 | 0404 | 0405 | 0506 | 0404 | 0505 | 1011 | 1414 | 0101 |
| 0505 | 0808 | 0304 | 0404 | 0404 | 0105 | 0404 | 0505 | 1010 | 1418 | 0102 |
| 0205 | 0808 | 0303 | 0404 | 0404 | 0507 | 0404 | 0509 | 1010 | 0718 | 0102 |
| 0206 | 0808 | 0303 | 0404 | 0405 | 0507 | 0404 | 0505 | 1212 | 0609 | 0102 |
| 0508 | 0809 | 0303 | 0404 | 0405 | 0508 | 0404 | 0505 | 0909 | 0716 | 0102 |
| 0505 | 0809 | 0303 | 0404 | 0404 | 0505 | 0202 | 0509 | 0910 | 2022 | 0101 |
| 0506 | 0810 | 0101 | 0404 | 0404 | 0517 | 0404 | 0509 | 0914 | 0710 | 0101 |
| 0104 | 0808 | 0304 | 0404 | 0102 | 1818 | 0202 | 0408 | 0510 | 1028 | 0202 |
| 0505 | 0808 | 0303 | 0404 | 0405 | 0105 | 0404 | 0509 | 1012 | 0920 | 0101 |
| *Population: Farm2* | | | | | | | | | | |
| 0407 | 0104 | 0606 | 0404 | 0406 | 0508 | 0406 | 0505 | 0911 | 2426 | 0101 |
| 0204 | 0404 | 0606 | 0404 | 0405 | 0712 | 0404 | 0709 | 1112 | 1016 | 0101 |
| 0405 | 0404 | 0607 | 0404 | 0404 | 0510 | 0404 | 0505 | 1111 | 1616 | 0101 |
| 0406 | 0404 | 0606 | 0404 | 0405 | 0707 | 0404 | 0505 | 0912 | 2830 | 0101 |
| 0406 | 0404 | 0606 | 0404 | 0404 | 0710 | 0404 | 0505 | 1112 | 2928 | 0101 |
| 0508 | 0404 | 0607 | 0304 | 0404 | 0520 | 0404 | 0709 | 1010 | 1230 | 0101 |
| 0406 | 0404 | 0606 | 0404 | 0405 | 0707 | 0404 | 0505 | 1212 | 2830 | 0101 |
| 0406 | 0404 | 0606 | 0304 | 0404 | 0710 | 0404 | 0505 | 0912 | 2830 | 0101 |
| 0406 | 0404 | 0606 | 0404 | 0405 | 0707 | 0404 | 0506 | 1112 | 2030 | 0101 |
| 0606 | 0404 | 0606 | 0404 | 0405 | 0505 | 0404 | 0505 | 0912 | 2028 | 0101 |
| 0307 | 0104 | 0709 | 0404 | 0405 | 0810 | 0204 | 0509 | 0910 | 1022 | 0102 |
| 0709 | 0406 | 0609 | 0404 | 0404 | 0610 | 0204 | 0404 | 0611 | 1014 | 0101 |
| 0607 | 0404 | 0607 | 0404 | 0404 | 0105 | 0404 | 0505 | 1012 | 0814 | 0101 |
| 0707 | 0404 | 0609 | 0304 | 0405 | 0808 | 0204 | 0505 | 0912 | 0710 | 0102 |
| 0607 | 0606 | 0609 | 0304 | 0404 | 1012 | 0204 | 0509 | 0911 | 0810 | 0102 |
| 0609 | 0404 | 0609 | 0404 | 0404 | 0510 | 0404 | 0505 | 0910 | 0710 | 0101 |
| 0505 | 0404 | 0606 | 0404 | 0404 | 0505 | 0404 | 0505 | 0909 | 1616 | 0101 |
| 0709 | 0404 | 0606 | 0404 | 0404 | 0512 | 0404 | 0509 | 1112 | 0708 | 0102 |
| 0607 | 0406 | 0609 | 0404 | 0404 | 0510 | 0404 | 0509 | 0913 | 1020 | 0101 |
| 0607 | 0404 | 0606 | 0404 | 0404 | 0505 | 0404 | 0505 | 1012 | 0707 | 0102 |
| 0707 | 0404 | 0607 | 0404 | 0305 | 0508 | 0404 | 0505 | 1212 | 1014 | 0101 |
| 0409 | 0406 | 0606 | 0405 | 0404 | 0505 | 0404 | 0505 | 1011 | 1420 | 0101 |
| 0607 | 0104 | 0609 | 0404 | 0404 | 0810 | 0405 | 0505 | 1013 | 0910 | 0102 |
| 0308 | 0404 | 0609 | 0404 | 0404 | 0808 | 0404 | 0505 | 1010 | 0607 | 0101 |
| 0609 | 0406 | 0707 | 0404 | 0405 | 0508 | 0404 | 0505 | 0910 | 1414 | 0101 |
| 0607 | 0104 | 0607 | 0404 | 0404 | 0608 | 0404 | 0509 | 0912 | 1214 | 0101 |
| 0209 | 0404 | 0606 | 0404 | 0404 | 0508 | 0204 | 0509 | 1112 | 0718 | 0102 |
| 0707 | 0104 | 0607 | 0404 | 0404 | 0508 | 0404 | 0505 | 0912 | 0914 | 0102 |
| 0505 | 0406 | 0607 | 0404 | 0505 | 0707 | 0404 | 0707 | 1010 | 0408 | 0101 |
| 0606 | 0404 | 0607 | 0404 | 0404 | 0508 | 0404 | 0607 | 0811 | 2431 | 0101 |
| 0608 | 0405 | 0707 | 0404 | 0404 | 0607 | 0404 | 0505 | 1011 | 0823 | 0101 |
| 0608 | 0406 | 0607 | 0404 | 0404 | 0707 | 0404 | 0505 | 0809 | 0606 | 0101 |
| 1010 | 0406 | 0609 | 0404 | 0506 | 0508 | 0404 | 0909 | 1010 | 0608 | 0101 |
| *Population: Farm3* | | | | | | | | | | |
| 0407 | 0404 | 0606 | 0505 | 0405 | 0305 | 0404 | 0809 | 1011 | 0809 | 0101 |
| 0406 | 0404 | 0606 | 0404 | 0405 | 0505 | 0406 | 0507 | 1111 | 0618 | 0101 |
| 0409 | 0404 | 0606 | 0404 | 0404 | 0508 | 0404 | 0910 | 1112 | 1016 | 0101 |
| 0709 | 0404 | 0606 | 0404 | 0505 | 0208 | 0404 | 0909 | 0910 | 0909 | 0101 |
| 0606 | 0404 | 0606 | 0404 | 0404 | 0808 | 0404 | 0505 | 1112 | 0610 | 0101 |
| 0407 | 0404 | 0606 | 0404 | 0404 | 0810 | 0404 | 0709 | 1012 | 0606 | 0101 |
| 0709 | 0404 | 0606 | 0405 | 0405 | 0508 | 0404 | 0509 | 1012 | 0120 | 0101 |
| 0508 | 0404 | 0606 | 0404 | 0405 | 0305 | 0404 | 0508 | 1011 | 1224 | 0101 |
| 0809 | 0404 | 0606 | 0404 | 0404 | 0510 | 0404 | 0509 | 0912 | 0608 | 0101 |
| 0709 | 0404 | 0606 | 0505 | 0405 | 0305 | 0404 | 0508 | 1212 | 0920 | 0101 |
| 0405 | 0405 | 0606 | 0404 | 0406 | 0305 | 0404 | 0507 | 1212 | 0628 | 0101 |
| 0505 | 0404 | 0606 | 0405 | 0405 | 0305 | 0404 | 0910 | 0910 | 1028 | 0101 |
| 0206 | 0404 | 0606 | 0404 | 0404 | 0808 | 0406 | 0508 | 0911 | 2026 | 0101 |
| 0507 | 0404 | 0606 | 0404 | 0405 | 1010 | 0404 | 0910 | 1111 | 0620 | 0101 |
| 0204 | 0404 | 0606 | 0404 | 0404 | 0508 | 0404 | 0909 | 0912 | 0626 | 0101 |
| 0306 | 0404 | 0606 | 0404 | 0405 | 0505 | 0404 | 0508 | 0909 | 0606 | 0202 |
| 0407 | 0404 | 0606 | 0405 | 0404 | 0507 | 0404 | 0505 | 1011 | 2529 | 0101 |
| 0909 | 0404 | 0606 | 0404 | 0405 | 0305 | 0404 | 0810 | 1112 | 0909 | 0101 |
| 0409 | 0406 | 0606 | 0405 | 0404 | 0707 | 0404 | 0809 | 1010 | 0910 | 0101 |
| 0707 | 0404 | 0606 | 0405 | 0405 | 0508 | 0404 | 0505 | 0809 | 1719 | 0101 |
| 0404 | 0404 | 0606 | 0505 | 0404 | 0507 | 0404 | 0505 | 1112 | 0627 | 0101 |
| 0407 | 0404 | 0606 | 0405 | 0404 | 0505 | 0404 | 0505 | 1012 | 1919 | 0101 |
| 0305 | 0404 | 0606 | 0404 | 0404 | 0310 | 0404 | 0509 | 1012 | 0609 | 0101 |
| 0405 | 0304 | 0606 | 0405 | 0404 | 0510 | 0404 | 0609 | 0909 | 1229 | 0101 |
| 0707 | 0404 | 0606 | 0405 | 0404 | 0305 | 0404 | 0509 | 1011 | 0929 | 0101 |
| 0709 | 0406 | 0606 | 0405 | 0404 | 0508 | 0404 | 0911 | 1111 | 0606 | 0101 |
| 0406 | 0406 | 0606 | 0404 | 0505 | 0505 | 0404 | 0909 | 0912 | 0918 | 0101 |
| 0909 | 0404 | 0606 | 0405 | 0404 | 0508 | 0404 | 0509 | 1111 | 1818 | 0101 |
| 0407 | 0404 | 0606 | 0405 | 0000 | 0508 | 0606 | 0204 | 0101 | 0208 | 0101 |
| 0709 | 0404 | 0606 | 0404 | 0404 | 0707 | 0404 | 0508 | 0912 | 1029 | 0101 |
| 0407 | 0304 | 0606 | 0405 | 0405 | 0505 | 0404 | 0809 | 0909 | 0628 | 0101 |
| 0607 | 0404 | 0606 | 0404 | 0404 | 0505 | 0404 | 0508 | 0912 | 1618 | 0101 |
| 0709 | 0404 | 0606 | 0404 | 0404 | 0505 | 0404 | 0909 | 0911 | 1828 | 0101 |
| 0507 | 0404 | 0606 | 0404 | 0404 | 0508 | 0404 | 0505 | 0000 | 0626 | 0101 |
| 0407 | 0304 | 0606 | 0405 | 0404 | 0307 | 0406 | 0507 | 0910 | 1218 | 0101 |
| 0409 | 0404 | 0606 | 0404 | 0404 | 0507 | 0404 | 0509 | 1212 | 0926 | 0101 |
| 0507 | 0404 | 0606 | 0404 | 0405 | 0508 | 0404 | 0508 | 1212 | 0606 | 0101 |
| 0408 | 0407 | 0606 | 0404 | 0406 | 0305 | 0404 | 0505 | 1010 | 0926 | 0101 |
| 0207 | 0404 | 0606 | 0404 | 0404 | 0308 | 0404 | 0809 | 0910 | 0922 | 0101 |
| 0909 | 0406 | 0606 | 0405 | 0405 | 0609 | 0406 | 0505 | 1012 | 0707 | 0101 |
| 0909 | 0404 | 0606 | 0405 | 0405 | 0508 | 0406 | 0507 | 1112 | 0628 | 0101 |
| 0304 | 0304 | 0607 | 0405 | 0505 | 0510 | 0404 | 0505 | 0912 | 0626 | 0101 |
| 0409 | 0405 | 0606 | 0404 | 0404 | 0508 | 0404 | 0809 | 0912 | 0609 | 0101 |
| 0509 | 0304 | 0606 | 0404 | 0404 | 0510 | 0406 | 0508 | 1011 | 0609 | 0101 |
| 0509 | 0404 | 0606 | 0404 | 0404 | 0512 | 0406 | 0909 | 1112 | 0909 | 0101 |
| 0406 | 0404 | 0606 | 0405 | 0404 | 0508 | 0404 | 0509 | 1012 | 0920 | 0101 |
| 0909 | 0303 | 0606 | 0404 | 0405 | 0505 | 0406 | 0915 | 0909 | 1014 | 0101 |
| 0404 | 0404 | 0606 | 0404 | 0405 | 0510 | 0404 | 0508 | 0811 | 0618 | 0101 |
| 0409 | 0404 | 0606 | 0404 | 0404 | 1010 | 0405 | 0509 | 0911 | 0609 | 0101 |
| 0507 | 0404 | 0606 | 0404 | 0405 | 0505 | 0404 | 0709 | 1011 | 0718 | 0101 |
| 0206 | 0404 | 0606 | 0404 | 0404 | 0508 | 0404 | 0808 | 0909 | 0622 | 0101 |
| 0607 | 0404 | 0606 | 0404 | 0405 | 0610 | 0406 | 0909 | 0909 | 1228 | 0101 |
| 0405 | 0404 | 0606 | 0404 | 0404 | 0210 | 0404 | 0509 | 1012 | 0620 | 0101 |
| 0607 | 0404 | 0606 | 0404 | 0404 | 0210 | 0404 | 0909 | 1012 | 0918 | 0101 |
| 0507 | 0404 | 0606 | 0404 | 0506 | 0505 | 0404 | 0509 | 0811 | 1228 | 0101 |
| 0507 | 0404 | 0606 | 0404 | 0404 | 0305 | 0404 | 0508 | 0911 | 1828 | 0101 |
| 0909 | 0404 | 0606 | 0404 | 0404 | 0710 | 0404 | 0509 | 0911 | 0928 | 0101 |
| 0509 | 0404 | 0606 | 0404 | 0404 | 0510 | 0406 | 0910 | 1112 | 0710 | 0101 |
| 0405 | 0306 | 0606 | 0404 | 0404 | 0508 | 0404 | 0910 | 0811 | 0618 | 0101 |
| 0407 | 0404 | 0606 | 0404 | 0404 | 0508 | 0404 | 0509 | 0909 | 2828 | 0101 |
| 0709 | 0405 | 0606 | 0104 | 0405 | 0612 | 0404 | 0809 | 1012 | 1828 | 0101 |
